# Approximating the Critical Domain Size of Integrodifference Equations

**DOI:** 10.1007/s11538-015-0129-x

**Published:** 2015-12-31

**Authors:** Jody R. Reimer, Michael B. Bonsall, Philip K. Maini

**Affiliations:** Mathematical and Statistical Sciences, 632 Central Academic Building, University of Alberta, Edmonton, AB T6G 2G1 Canada; Mathematical Ecology Research Group, Department of Zoology, University of Oxford, Tinbergen Building, South Parks Road, Oxford, OX1 3PS UK; Wolfson Centre for Mathematical Biology, Mathematical Institute, University of Oxford, Andrew Wiles Building, Radcliffe Observatory Quarter, Woodstock Road, Oxford, OX2 6GG UK

**Keywords:** Integrodifference equations, Approximation, Critical domain size, Bifurcation point

## Abstract

Integrodifference (IDE) models can be used to determine the critical domain size required for persistence of populations with distinct dispersal and growth phases. Using this modelling framework, we develop a novel spatially implicit approximation to the proportion of individuals lost to unfavourable habitat outside of a finite domain of favourable habitat, which consistently outperforms the most common approximations. We explore how results using this approximation compare to the existing IDE results on the critical domain size for populations in a single patch of good habitat, in a network of patches, in the presence of advection, and in structured populations. We find that the approximation consistently provides results which are in close agreement with those of an IDE model except in the face of strong advective forces, with the advantage of requiring fewer numerical approximations while providing insights into the significance of disperser retention in determining the critical domain size of an IDE.

## Introduction

Integrodifference equations (IDEs) are an established way to model populations in which growth does not occur simultaneously with dispersal. They are discrete in time, continuous in space, and in the simplest instances, IDEs behave like reaction–diffusion equations (Fisher 1937). In the event that the growth dynamics are overcompensatory, IDEs can exhibit complicated behaviour such as periodic solutions or chaotic dynamics (Kot 1992).

Initially used to model the propagation of alleles (Slatkin 1973, 1975), IDEs were first applied to population biology by Kot and Schaffer (1986). Since then, they have been analysed for travelling wave solutions (Hsu and Zhao 2008; Kot 1992; Kot et al. 1996; Neubert and Parker 2004; Weinberger et al. 2008) and dispersal-driven instability in predator–prey interactions (Neubert et al. 1995), and the effects of different dispersal strategies have been considered in both spatially and temporally varying habitats (Hardin et al. 1990). They have also been used to model shifting species ranges under climate change (Zhou and Kot 2011). Much of this work has been done in the context of plant growth and dispersal (Andersen 1991; Bullock et al. 2012) and for marine populations (Botsford et al. 2001).

Here, we outline the conventional IDE framework in order to familiarize the reader with the basic assumptions and notation. We first consider the growth of a non-spatial population according to a function $f(N_t)$ where $N_t$ is the density of the female population at time $t \ge 0$. This will typically be a nonlinear function and can often be written as $f(N_t) = N_t g(N_t)$, where $g(N_t)$ is the per capita growth rate. For a population modelled in discrete time with no movement,1with a given initial condition $N_{t_0} = N_0$ for some $t_0 \ge 0$ and $N_0 \ge 0$. A short list of commonly used growth functions is included in Table 1. Note that our modelling efforts will focus solely on females, which corresponds to the assumption that the entire population reproduces according to the chosen growth function. IDEs incorporate a spatial component via a dispersal kernel, a probability density function (PDF) here denoted *k*(*x*, *y*), which is the probability that an individual starting at point *y* will settle at point *x* by the next time step. We here consider a one-dimensional domain for simplicity, though we discuss the implications of this work for higher-dimensional domains in Sect. 5. In many situations, this is not biologically unreasonable; for example, many marine species live in the shallower waters near the coast, and the scale of any successful dispersal will be much greater along the coast than out towards open water. By integrating the dispersal kernel over the domain of interest, the number of individuals which move to point *x* over the subsequent time step can be determined. Since we are interested in populations on a finite domain, we will typically have

2 $$\begin{aligned} \int _{\varOmega }{k(x,y)\mathrm{d}y} < 1, \end{aligned}$$

where $\varOmega$ is the spatial domain relevant to the question of interest. Our central question is focused on the critical domain size. The idea of a critical domain size is a common notion in population ecology, first considered in Skellam (1951) and Kierstead and Slobodkin (1953). It addresses the question of how large a domain needs to be in order to sustain a population in the absence of immigration, given hostile conditions outside the domain. Mathematically, we will define this to be the smallest domain size for which there exists a stable non-zero steady state. We initially consider the critical domain size of a single domain of length *L*, so that $\varOmega = [-L/2, L/2]$. Combining the original growth model (now allowing for spatial dependence of the growth function) with the dispersal kernel results in

3 $$\begin{aligned} N_{t+1}(x) = \int _\varOmega {k(x,y)f(N_t(y);y)\mathrm{d}y}, \end{aligned}$$

though for other formulations, see Lutscher (2008).

### Dispersal Kernels

Empirical research on dispersal patterns has resulted in the realization that dispersal events may take a range of probability distributions, and it is unrealistic to assume that isotropic diffusion captures the wide range of observed dispersal behaviour. A possible simplification of dispersal processes can be made from first principles to describe dispersal as a mechanistically derived PDF (Guichard et al. 2004; Mollison 1977). Dispersal kernels can either be phenomenological (derived from statistical analysis of data) or mechanistic (derived from underlying assumptions about the biological process). Phenomenological models can be very useful in simulation, as algorithms allow random numbers to be drawn from well-known distributions. If we are interested, however, in how environmental factors (e.g. seasonal winds or currents) may affect dispersal distances, mechanistic models may better aid in gaining understanding (Jongejans et al. 2008).

Several dispersal kernels have been derived based on various mechanistic assumptions (see Lutscher et al. 2005 for a clear explanation of the derivation procedure). Models with synchronous settling lead to the Gaussian (or normal) and Cauchy distributions (Jongejans et al. 2008; Neubert et al. 1995). The Laplace and fat-tailed distributions arise when organisms settle at a constant rate (Lutscher et al. 2005). If the settling rate increases or decreases as a power of time, we obtain the double Weibull distribution, and if the settling rate tends monotonically towards a constant, the double Gamma distribution is obtained (see Neubert et al. 1995 for details). In order to isolate the shapes of these distributions for comparison, we scale them using the mean of the strictly positive distribution (i.e. the mean of *k*(*x*) for $x \ge 0$). Table 1 provides a convenient reference to the kernels used in this work.

**Table 1:** Commonly used growth functions and dispersal kernels and their key properties

*Discrete growth functions*		
Logistic	$$N_{t+1} = {\left\{ \begin{array}{ll}rN_t\left( 1 - N_t\right) , &{} N_t \in [0,1]\\ 0, &{} N_t > 1 \end{array}\right. }$$	Overcompensatory (May 1976; Maynard Smith 1968)
Ricker	$N_{t+1} = N_t\exp {\left\{ r\left( 1 - N_t\right) \right\} }$	Overcompensatory (Ricker 1954)
Beverton–Holt	$N_{t+1} = \frac{rN_t}{1 + N_t(r-1)}$	Compensatory (Beverton and Holt 1957)
*Dispersal kernels*		
Gaussian	$k(x,y) = \frac{\exp {(-(x-y)^2/2\sigma ^2)}}{\sigma \sqrt{2\pi }}$	Mean: $\sigma \sqrt{2/\pi }$ (Lockwood et al. 2002)
Laplace	$k(x,y) =$ $\sqrt{\frac{\alpha }{4D}}\exp {\left( -|x-y|\sqrt{\frac{\alpha }{D}}\right) }$	Mean: $\sqrt{\frac{D}{\alpha }}$ (Kot 1992; Lockwood et al. 2002)
Cauchy	$k(x,y) = \left\{ \pi b\left( 1 + \left[ \frac{(x-y)}{b}\right] ^2\right) \right\} ^{-1}$	Mean: undefined, conventionally $b = 1$ for scaling purposes (Jongejans et al. 2008)
Fat-tailed	$$ k(x,y) =$$ $$ \theta \mathfrak {R}\left\{ \hbox {E}_1(\imath \theta |x-y|)\exp {(\imath \theta |x-y|)}\right\} /\pi \nonumber $$	Mean: undefined, $$\theta = \alpha /\rho $$ and $$\hbox {E}_1$$ is the exponential integral (Lutscher et al. 2005)

## Approximations to an IDE

Integrodifference equations are often not analytically tractable, and so have to be simulated numerically, which makes it difficult to compare the dependence on parameters of the critical domain sizes of IDEs with different dispersal kernels (Slone 2011). It is desirable to determine a good approximation to this modelling framework in order to exploit its structure of discrete time and continuous space without needing to find exact solutions.

An IDE has the same bifurcation structure as its growth function $f(N_t)$, as determined by its linearized growth rate *r* (or $\mathrm {e}^r$, Table 1), but the bifurcation value $r^*$ is greater because of individuals lost to the unfavourable habitat outside the domain. As the size of the domain tends to infinity, the bifurcation value of *r* tends to that of the non-spatial growth function. If we are unconcerned with the population’s spatial distribution inside the domain, we can approximate the loss of individuals to unfavourable areas by simply multiplying the growth function by some number, say $A \le 1$, so that

4 $$\begin{aligned} \bar{N}_{t+1} = Af(\bar{N}_t), \end{aligned}$$

where $\bar{N}_t$ denotes the total population inside the domain. Given that the growth functions considered in this work are of the form $f(N_t) = rN_tg(N_t)$, where *r* is the intrinsic rate of growth, we can see that changing *A* results in the same behaviour as changing *r* in the non-spatial growth function. Note that *A* need not change linearly as the length of the domain changes.

### Previous Approximations to an IDE

Van Kirk and Lewis (1997) developed an approximation for *A* using the *dispersal success function* and the *average dispersal success function*. The dispersal success function *s*(*y*) describes the probability that an individual starting at point *y* settles within the domain $\varOmega$ over the next time step, as given by

5 $$\begin{aligned} s(y) = \int _\varOmega {k(x,y)\mathrm{d}x}. \end{aligned}$$

The average dispersal success over the domain is then

6 $$\begin{aligned} S = \frac{1}{|\varOmega |}\int _\varOmega {s(y)\mathrm{d}y}. \end{aligned}$$

Note that *S* will depend on the parameters of $\varOmega$; in our case, $S = S(L)$ where *L* is the domain length. Van Kirk and Lewis (1997) used this as a spatially implicit approximation to an IDE of the form

7 $$\begin{aligned} N_{t+1} = Sf(N_t), \end{aligned}$$

where *S* now serves to approximate the proportion of individuals remaining inside the domain after the dispersal phase. This spatially implicit model had the same qualitative behaviour as the growth function $f(N_t)$, but was scaled by $0 \le S \le 1$. The analysis of the IDE can be reduced to the analysis of a simpler difference equation if this average dispersal success rate is known, and the first-order approximate solution to the equilibrium population size $N^*$ proposed in Van Kirk and Lewis (1997) is the solution to the algebraic equation

8 $$\begin{aligned} N^* = S f(N^*). \end{aligned}$$

If the growth function $f(N^*)$ bifurcates away from the zero solution to a stable non-zero solution as some parameter is varied, this behaviour is now scaled by *S*. For example, the logistic map (Table 1) bifurcates away from zero at $r = 1$ and so (7) bifurcates at $Sr = 1$. Since $S = S(L)$, this can be used to approximate the critical domain size $L^*$ at which this occurs.

As discussed by Van Kirk and Lewis (1997), *S* most closely approximates the steady state solution when the equilibrium solution is close to the spatially averaged solution, i.e. when *s*(*x*) is similar to the steady state solution (Van Kirk and Lewis 1997). They also observed, using numerics, that the dispersal success function provides a reasonable approximation to the population’s long-time distribution over its patch, given a suitable domain size and growth function, and it is this idea that we use to develop an improved approximation and thus gain a more accurate approximation of the critical domain size.

## The Modified Dispersal Success Approximation, $\mathbf {\widehat{S}}$

While the average dispersal success function provides a rough estimate of the fraction of individuals which stay within the domain from one time step to the next, it assumes a uniform distribution of individuals throughout the patch. A better approximation to the true fraction of dispersing individuals which are retained could be obtained if we could weight the dispersal success values by the proportion of the population at each point, so that

9 $$\begin{aligned} S_{\mathrm{mod}} = \frac{1}{|\varOmega |}\int _{\varOmega }{\left( \frac{N_t(y)}{\frac{1}{|\varOmega |}\int _{\varOmega }{N_t(z)\mathrm{d}z}}\right) s(y)\mathrm{d}y}. \end{aligned}$$

This formulation requires us to know $N_t(y)$, which negates the purpose of having an approximation to the population in the first place. Instead, we will approximate the shape of the population by the dispersal success function at each point to obtain a novel approximation, which we will now refer to as the *modified average dispersal success* function $\widehat{S}$. Substituting *s*(*y*) into (9) for $N_t(y)$ results in

10 $$\begin{aligned} \widehat{S} = \frac{1}{|\varOmega |}\int _{\varOmega }{\left( \frac{s(y)}{\frac{1}{|\varOmega |}\int _{\varOmega }{s(z)\mathrm{d}z}}\right) s(y)\mathrm{d}y} = \frac{1}{|\varOmega |}\int _{\varOmega }{\left( \frac{s(y)}{S}\right) s(y)\mathrm{d}y}. \end{aligned}$$

It can be shown (Appendix 1) that this modified approximation $\widehat{S}$ predicts equivalent or larger average dispersal success than the approximation of Van Kirk and Lewis (1997) for the kernels and domain considered. This is because the approximation weights the (higher) dispersal success of individuals in areas expected to have more individuals [i.e. for higher values of *s*(*y*)] more heavily than the dispersal success of those with low *s*(*y*) values when calculating average dispersal success. The reasoning for this relies on the symmetry of the kernels; locations with high dispersal success will, in turn, have more individuals from other locations settle there.

### Illustrative Example

We now look at a simple example using the Laplace dispersal kernel, which we have chosen in order to enable direct comparison between the approximations and the analytic expression for the critical domain length, since the Laplace kernel provides one of the few examples in which we can find an analytic expression for both. For an IDE with a Laplace dispersal kernel and logistic growth function (Table 1), we followed the method of Kot and Schaffer (1986) and obtained an explicit solution for the critical domain length $L^*$

11 $$\begin{aligned} L^* = \frac{2\, b}{\sqrt{r-1}}\tan ^{-1}{\left( \frac{1}{\sqrt{r-1}}\right) }, \end{aligned}$$

where $b = \sqrt{\frac{D}{\alpha }}$. Details of this derivation can be found in Standard IDE Example section of Appendix 2. Following (5) and (6), the dispersal success function at a given point *y* is

12 $$\begin{aligned} s(y) = 1 - 2\, \mathrm {e}^{-\left( \frac{L}{2} + y\right) /b} - 2\, \mathrm {e}^{\left( y - \frac{L}{2}\right) /b} \end{aligned}$$

and the average dispersal success is

13 $$\begin{aligned} S = 1 + \frac{b\left( \mathrm {e}^{-L/b} - 1\right) }{L}. \end{aligned}$$

The logistic growth function experiences a bifurcation in the stable steady state at $r = 1$ in the non-spatial model, so for this approximation, the bifurcation now occurs at $Sr = 1$. Thus, the approximation to the critical domain length $L^*_S$ is the solution to

14 $$\begin{aligned} S = 1 + \frac{b\left( \mathrm {e}^{-L/b} - 1\right) }{L} = \frac{1}{r}. \end{aligned}$$

The corresponding modified dispersal success approximation for the Laplace kernel from Eq. (10) is

15 $$\begin{aligned} \widehat{S} = \frac{2\, L\, \mathrm {e}^{\frac{L}{b}} - b + 4\, L\, \mathrm {e}^{\frac{2\, L}{b}} + 8\, b\, \mathrm {e}^{\frac{L}{b}} - 7\, b\, \mathrm {e}^{\frac{2\, L}{b}}}{4\, \mathrm {e}^{\frac{L}{b}}\, \left( b + L\, \mathrm {e}^{\frac{L}{b}} - b\, \mathrm {e}^{\frac{L}{b}}\right) }, \end{aligned}$$

and so the domain length $L^*_{\widehat{S}}$ at which the solution bifurcates, using $\widehat{S}$ as the approximation to *A* in (4), can be found by solving for *L* in

16 $$\begin{aligned} \widehat{S} = \frac{2\, L\, \mathrm {e}^{\frac{L}{b}} - b + 4\, L\, \mathrm {e}^{\frac{2\, L}{b}} + 8\, b\, \mathrm {e}^{\frac{L}{b}} - 7\, b\, \mathrm {e}^{\frac{2\, L}{b}}}{4\, \mathrm {e}^{\frac{L}{b}}\, \left( b + L\, \mathrm {e}^{\frac{L}{b}} - b\, \mathrm {e}^{\frac{L}{b}}\right) } = \frac{1}{r}. \end{aligned}$$

**Fig. 1:**
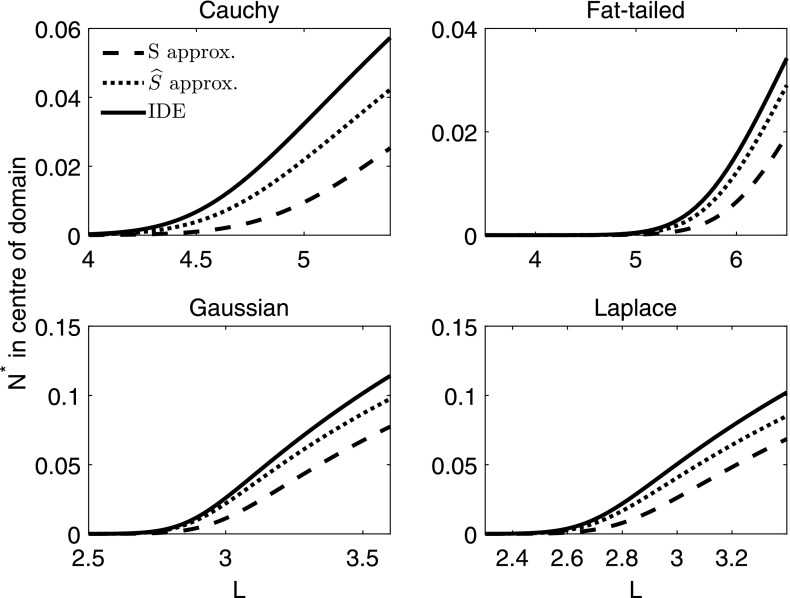
Stable steady states for four mechanistic dispersal kernels and varying domain lengths. Each IDE and approximation are subject to logistic growth with growth rate $r = 1.5$, and other parameters are as follows: for the Cauchy distribution, $b = 1$, for the fat-tailed distribution, $\alpha = 1.2$, $\rho = 2$, for the Gaussian distribution, $\sigma = \sqrt{\pi /2}$, and for the Laplace distribution, $\alpha = 1$, $D = 1$. We can see here that in each case, the stable steady state solution of the new approximation ($\widehat{S}$) is much nearer to that of the IDE than the average dispersal success approximation (*S*)

**Fig. 2:**
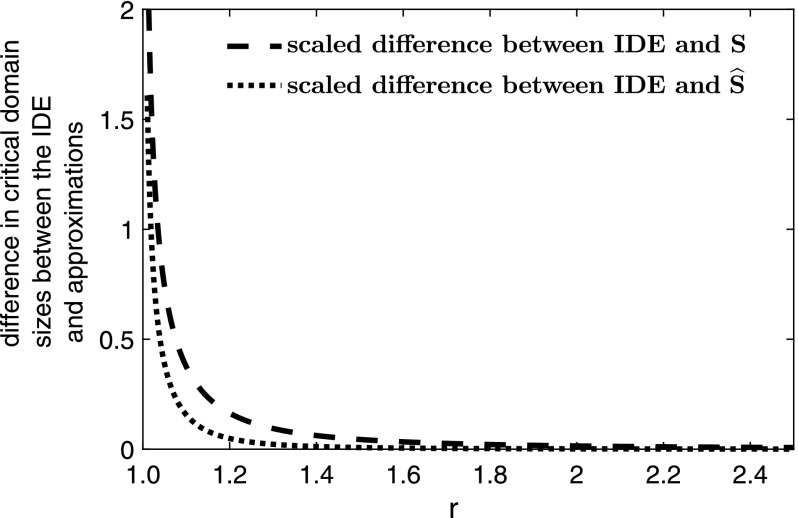
Difference between the critical domain lengths of an IDE with logistic growth and a Laplace kernel ($\alpha = D = 1$), and the two approximations *S* and $\widehat{S}$, scaled to value of $L^*$ as determined by the IDE. Here, we can see that for *r* values close to 1, there is the greatest difference between the critical domain size predicted by the IDE and the two approximations. It is also for *r* values close to 1 that the new $\widehat{S}$ approximation most significantly outperforms the *S* approximation

Simulations confirm that $\widehat{S}$ consistently provides a closer approximation to the critical domain size $L^*$ than *S*, and this holds for a variety of kernels and varying values of *r* (Figs. 1, 2). The difference between the two approximations decreases for increasing values of *r*, and this is most significant for values of *r* slightly larger than 1, which is also the parameter regime where the two approximations perform worst (Fig. 2).

## Extended Applications of $\mathbf {\widehat{S}}$

### Network of Domain Patches

Often, a single finite patch will not accurately represent the domain of interest. For example, anthropogenic landscape fragmentation may require a population to exist on multiple small patches instead of one larger one. In marine reserve design, it is commonly thought to be more beneficial to have multiple smaller reserves than one large one (Gaines et al. 2012; Nowlis and Roberts 1999; Palumbi 2001; Roberts et al. 2003). We will call a set of patches connected chiefly via the dispersal of mobile individuals a network of patches. One way this has been modelled is through an idealized structure, with an infinite network of patches of all the same size (having width *w*) and equally spaced (with periodicity *L*). A simple mathematical interpretation allowing for some analysis is to have “good” and “bad” habitat patches, as conceived of in Shigesada et al. (1986). This spatially periodic growth function has been modelled in Lutscher (2008) and Shigesada et al. (1986) as

17 $$\begin{aligned} f(N(x);x) = {\left\{ \begin{array}{ll} f_1(N(x);x), &{} x\in [nL-w/2,\, nL+w/2], \ n \in \mathbb {Z} \\ f_2(N(x);x), &{} x\in (nL+w/2,\, (n+1)L-w/2), \ n \in \mathbb {Z} \end{array}\right. } \end{aligned}$$

assuming that $[nL - w/2,nL + w/2]$, $n \in \mathbb {Z}$, are the patches of good habitat, and that $[nL + w/2,\,(n+1)L- w/2]$, $n \in \mathbb {Z}$, are the hostile areas. Under assumptions of a very hostile environment between patches, we set $f_2 = 0$. As before, we will suppose *f*(*N*(*x*); *x*) is of the form $f(N(x);x) = N(x) g(N(x);x)$ where $g(\cdot ;x)$ is now periodic in *x* with period *L*.

#### Approximation on a Network of Patches

We will now attempt to approximate the relationship between the width and spacing of patches in order to determine which configurations have a stable non-zero steady state. The first approximation to *A* in (4) that we will mention here for a network of patches is perhaps the most intuitive (Dewhirst and Lutscher 2009). As found in Dewhirst and Lutscher (2009), we could take *A* to be the fraction of favourable habitat out of all available locations, i.e. $A = w/L$. We could also use the average dispersal success function for *A* (Lutscher and Lewis 2004). For an infinite patch network, Lutscher and Lewis (2004) have shown that the average dispersal success for a patch, and thus, by symmetry, for every patch, is

18 $$\begin{aligned} S = \frac{1}{w}\int _{-w/2}^{w/2}{s(y)\mathrm{d}y}, \end{aligned}$$

where

19 $$\begin{aligned} s(y) = \sum _{n = -\infty }^{\infty }{\int _{nL-w/2}^{nL+w/2}{k(x,y)\mathrm{d}x}}. \end{aligned}$$

By the same reasoning as before, we consider a modified average dispersal success function of the form

20 $$\begin{aligned} \widehat{S} = \frac{1}{w}\int _{-w/2}^{w/2}{\frac{s^2(y)}{S}\mathrm{d}y}. \end{aligned}$$

This is again based on the assumption that *s*(*y*), the dispersal success approximation at each point, adequately approximates the population density.

#### Example of a Network of Patches

**Fig. 3:**
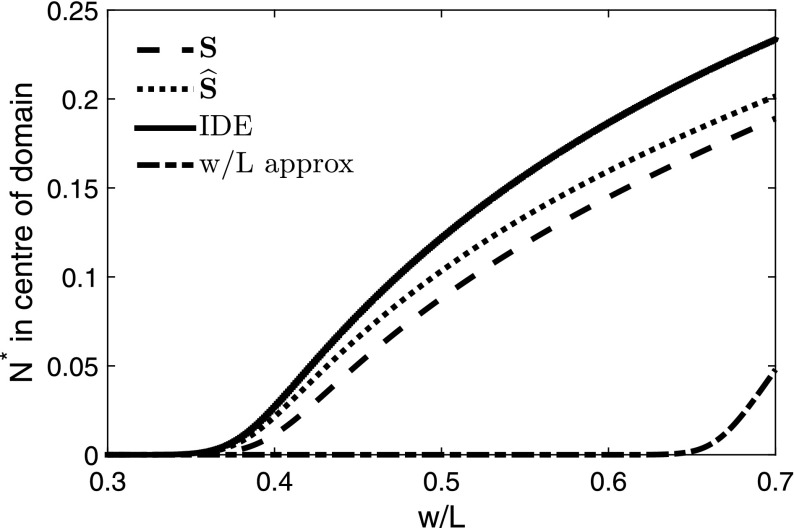
Stable steady state of the IDE and three approximations of Sect. 4.1.2 for varying fractions of good patches. The IDE has a Laplace kernel and logistic growth with $r = 1.5$ and an initial condition of 0.5 everywhere in the domain. We vary *w*, keeping *L* constant at 7 times the mean dispersal distance. A similar figure is produced if we vary *L* and keep *w* constant. Zero is a steady state throughout, but loses stability to the non-trivial steady state for large enough values of *w* / *L*

For an analytically tractable illustrative example, we choose the Laplace kernel and logistic growth and follow the method outlined by Van Kirk and Lewis (1997) (see Example on a Network of Patches section of Appendix 2 for details). The stable steady state solution to the IDE changes from the zero steady state to a non-zero steady state when the following relationship (see Fig. 3) is satisfied:

21 $$\begin{aligned}&\, 4G - \exp {(L - \textit{LR})}\sin {(\textit{GLR})} + \exp {(\textit{LR} - L)}\sin {(\textit{GLR})} \nonumber \\&- G^2\exp {(\textit{LR} - L)}\sin {(\textit{GLR})} - 2G\exp {(L - \textit{LR})}\cos {(\textit{GLR})} \nonumber \\&- 2G\exp {(\textit{LR} - L)}\cos {(\textit{GLR})} + G^2\exp {(L - \textit{LR})}\sin {(\textit{GLR})} = 0, \end{aligned}$$

where $G = \sqrt{r-1}$, *R* is the fraction of domain in a good patch, and *L* is the periodicity. In order for this stable non-trivial solution to be real, *r* must be greater than 1 so that *G* is real and positive. Recall that *r* is the linearized growth rate of the non-spatial growth model, so $r > 1$ also implies that the population is doing more than just replenishing itself in the good patches. Van Kirk and Lewis (1997) highlighted that as *L* tends to infinity, the relationship between *R* and *L* tends asymptotically to the critical domain size for a single patch divided by the periodicity. Intuitively, for small periodicities, connectivity between patches is important for population survival, but once the patches get too far apart, each individual one must retain sufficient young produced by its own population in order to persist.

We will now compare the bifurcation values of the approximations to those of the IDE. In this example, the dispersal success function is

22 $$\begin{aligned} s(y) = \frac{\mathrm {e}^{\frac{y}{b}}\, \left( \mathrm {e}^{\frac{w}{b}} - 1\right) }{2\, \mathrm {e}^{\frac{w}{2\, b}}\, \left( \mathrm {e}^{\frac{L}{b}} - 1\right) } - \frac{1}{2\, \mathrm {e}^{\frac{w + 2\, y}{2\, b}}} - \frac{1}{2\, \mathrm {e}^{\frac{w - 2\, y}{2\, b}}} + \frac{\left( \mathrm {e}^{\frac{w}{b}} - 1\right) }{2\, \mathrm {e}^{\frac{y}{b}}\, \mathrm {e}^{\frac{w}{2\, b}}\, \left( \mathrm {e}^{\frac{L}{b}} - 1\right) } + 1, \end{aligned}$$

and the average dispersal success *S* for a given patch is

23 $$\begin{aligned} S = \frac{w + b\, \left( \mathrm {e}^{\frac{-w}{b}} - 1\right) + \frac{b\, {\left( \mathrm {e}^{\frac{w}{b}} - 1\right) }^2}{\mathrm {e}^{\frac{w}{b}}\, \left( \mathrm {e}^{\frac{L}{b}} - 1\right) }}{w}. \end{aligned}$$

The modified average dispersal success $\widehat{S}$ for a patch is

24 $$\begin{aligned} \begin{aligned} \widehat{S}&= \left[ \frac{1}{\left( (w-b)\mathrm {e}^{\frac{2L + 2w}{b}} - b\mathrm {e}^{\frac{L + w}{b}} + (b+w)\mathrm {e}^{\frac{2w}{b}} - b\mathrm {e}^{\frac{3w}{b}} + b\mathrm {e}^{\frac{2L + w}{b}} + b\mathrm {e}^{\frac{L + 3w}{b}} - 2w\mathrm {e}^{\frac{L + 2w}{b}}\right) }\right] \\&\quad \;\times \left[ (4w-7b)\mathrm {e}^{\frac{2L + 2w}{b}} - 6b\mathrm {e}^{\frac{L + w}{b}} - b\mathrm {e}^{\frac{2L}{b}} + (7b + 4w)\mathrm {e}^{\frac{2\, w}{b}} + (2w - 8b)\mathrm {e}^{\frac{3w}{b}} + b\mathrm {e}^{\frac{4w}{b}}\right. \\&\quad \; \left. + \,(8b + 2w)\mathrm {e}^{\frac{2L + w}{b}} + 6b\mathrm {e}^{\frac{L + 3w}{b}} - 12w\mathrm {e}^{\frac{L + 2w}{b}} \right] \left( \frac{1}{4}\right) . \end{aligned}\nonumber \\ \end{aligned}$$

The stable steady state resulting from these approximations, as well as from the IDE, are shown in Fig. 3. Taking the limit of (24) as $L \rightarrow \infty$, the modified average dispersal success for a single patch results as a special case.

**Fig. 4:**
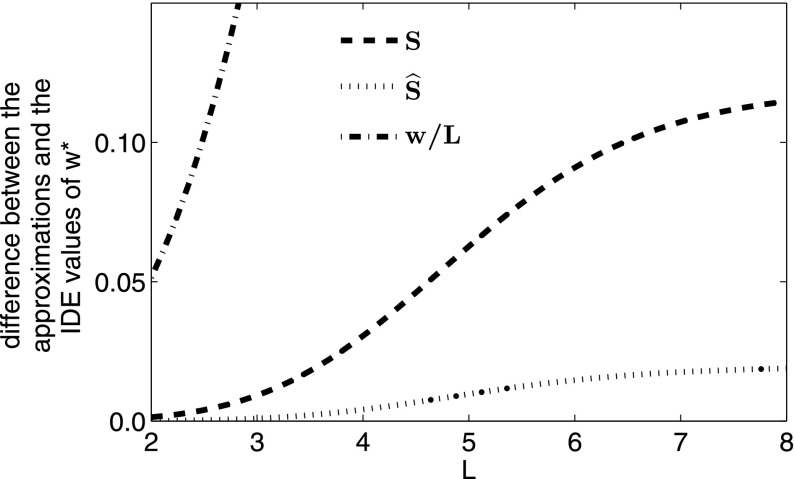
The difference between the bifurcation value of $w^*$ predicted by the three approximations to an IDE with a Laplace kernel and logistic growth, compared with the bifurcation value of the IDE. The growth rate *r* in the good patches is 1.5, and both *w* and *L* are scaled to the mean dispersal distance of the Laplace kernel

**Fig. 5:**
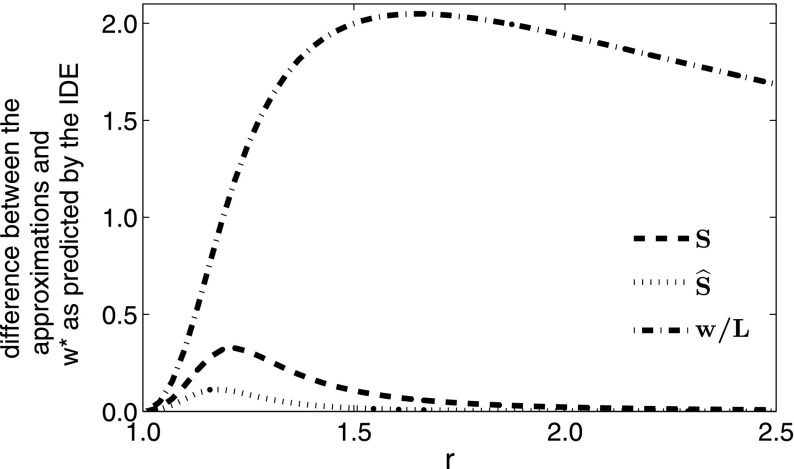
Difference between the IDE bifurcation value $w^*$ and those predicted by the three approximations $\widehat{S}$, *S*, and *w* / *L* for varying values of *r*. *L* and *w* are scaled to the mean dispersal distance for $L = 7$. Observe that for values of *r* between 1 and 2, the approximations do not perform as well as for other values of *r*, but the modified approximation $\widehat{S}$ still is a better approximation to the IDE than either *w* / *L* or *S*

As the good patches become further apart (i.e. we increase *L* in Fig. 4), the width $w^*$ necessary to sustain a population becomes larger, with the eventual limit of $w^* \rightarrow L^*$, the critical domain size of a single patch with the same demographic parameters. In Fig. 4, the new dispersal success approximation $\widehat{S}$ again outperforms *S* exactly where this matters most, i.e. where they both perform most poorly, which in this case is for large values of *L*, where $w^*$ is close to $L^*$. If we instead vary *w* and fix *L*, the corresponding trend is observed, i.e. as *w* tends to the critical domain size $L^*$ for a single patch, the periodicity *L* tends towards infinity.

We now consider the effects of varying *r* on the closeness of the approximations to the IDE value. In Fig. 5, for values of *r* greater than approximately 1.5, both *S* and $\widehat{S}$ provide a very close approximation to the IDE. As we decrease *r* from 1.5, the approximations start to fail, but as we get very close to $r = 1$, the fact that the population cannot persist on a domain of any size if there is any loss due to dispersal dominates the other mechanisms at play.

Thus, all three of the approximations, as well as the IDE, have critical domain lengths tending to infinity as *r* tends to 1. $\widehat{S}$ remains a better approximation than *S*, and this is again most evident where the two approximations are the furthest from the true solution.

**Fig. 6:**
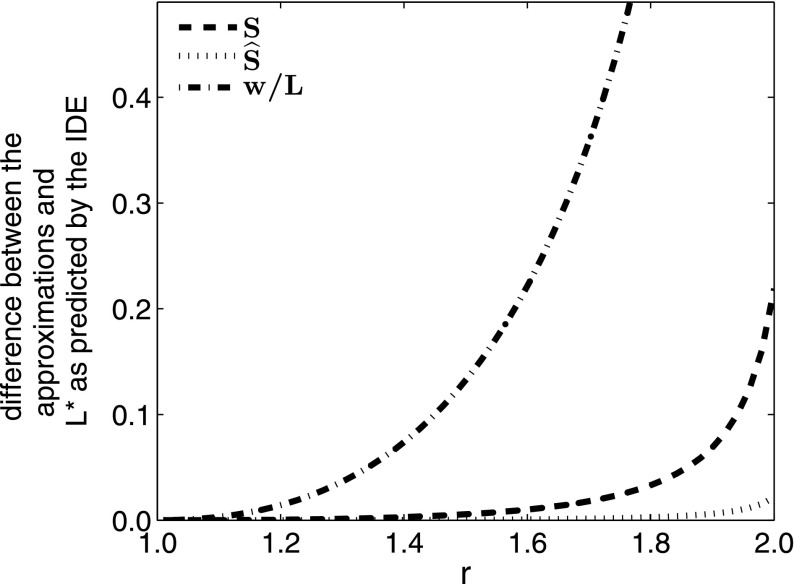
Difference between the IDE bifurcation value $L^*$ and the critical value for *L* predicted by the three approximations $\widehat{S}$, *S*, and *w* / *L*. *L* and *w* are scaled to the mean dispersal distance with $w = 1.5$. Observe that for increasing values of *r*, the approximations fail, but the modified approximation $\widehat{S}$ is much closer to the IDE here than either the *w* / *L* approximation or the *S* approximation

In Fig. 6, for a given width, $\widehat{S}$, *S* and *w* / *L* provide the best approximations for smaller values of *r* and, as before, $\widehat{S}$ most strongly outperforms *S* and *w* / *L* where they are both the weakest, which here is for larger *r* values.

### Stage-Structured Models

While IDEs capture the fact that many species do not reproduce and disperse simultaneously, it is often not biologically accurate to assume that first *all* individuals reproduce and then *all* individuals, both old and young, disperse in the same way. We know that for many marine species of interest, only the juveniles disperse and only those that have been recruited to the population can reproduce. Similarly for plants, seeds are subject to dispersal and plants which have reached reproductive maturity remain in one location.

Matrix population models are a well-established tool in population biology, and stage-structured models are especially appropriate for these populations, where fecundity is often not dependent on age but rather on size or stage. Neubert and Caswell (2000) first combined matrix population models with IDEs to create integrodifference matrix population models (IMP models). IMP models are formulated by combining population growth dynamics (now for a population with *m* stages) with the dispersal behaviour of each stage. Here, $\mathbf {N}_t$ denotes an $(m \times 1)$ population density vector and the growth dynamics are governed by

25 $$\begin{aligned} \mathbf {N}_{t+1}(x) = \mathbf {R}(\mathbf {N}_t(x))\mathbf {N}_t(x). \end{aligned}$$

$\mathbf {R}$ is an $(m\times m)$ transition matrix where entry $R_{ij}$ denotes the rate at which individuals in stage *j* at time *t* produce individuals of stage *i* by time $t+1$ (Caswell 2006). If we wish to account for varying conditions in the domain, we can also allow these entries to depend on space, i.e. $\mathbf {R}(\mathbf {N}_t(x); x)$ (for a comprehensive look at matrix population models, see Caswell 2006). Dispersal is included by integrating an $(m\times m)$ matrix $\mathbf {K}$ of dispersal kernels over the domain. Each entry $k_{ij}(x,y)$ of $\mathbf {K}$ denotes the probability that an individual who makes the transition from stage *j* to stage *i* during the time step also moves from location *y* to location *x* in that time step (Neubert and Caswell 2000). In order to maintain our assumption of sedentary adults, $k_{ij}$ will be the Dirac delta function

26 $$\begin{aligned} \delta (x,y) = {\left\{ \begin{array}{ll} + \infty , &{} |x-y| = 0\\ 0, &{} |x-y| \ne 0 \end{array}\right. } \end{aligned}$$

for adult stages. Combining these two components results in the IMP model

27 $$\begin{aligned} \mathbf {N}_{t+1}(x) = \int _{\varOmega }[\mathbf {K}(x,y)\circ \mathbf {R}(\mathbf {N}_t(y);y)]\mathbf {N}_t(y)\mathrm{d}y \end{aligned}$$

where $\circ$ represents element-by-element multiplication rather than standard matrix multiplication (Neubert and Caswell 2000). Just like an IDE, these models can exhibit a wide range of behaviour, including periodic cycles and chaos if elements of the transition matrix include nonlinearities. IMP models require us to find the eigenvalues of matrices, rather than of the IDE integral operator (Neubert and Parker 2004). Lutscher and Lewis (2004) and, more recently, Robertson and Cushing (2012), have analysed the critical domain size problem for these models. We will here use some of their results and then adapt our approximation to fit this framework and compare it with other approximations currently available (Lutscher and Lewis 2004; Robertson and Cushing 2012).

The critical domain size for IMP models is determined in a similar way as it was in the case of IDEs without stage structure. This involves looking for where the stable steady state solution bifurcates from the zero solution to a non-trivial solution. See Stage-Structured Population Theory section of Appendix 2 for a brief overview of the general theory for matrices.

#### Approximations for Structured Populations

A stage-structured modification to the average dispersal success function was carried out by Lutscher and Lewis (2004), and here we show that our modified average dispersal success function outperforms this approximation. They define a matrix of dispersal success $\mathbf {K}(x,y)$, where for each $k_{ij}(x,y)$, they determined $s_{ij}(y)$ which is the probability that an individual of stage *i* who was produced by an individual in stage *j* at location *y* settles in the domain of interest $\varOmega$. The matrix of dispersal success functions for a given point *y* is

28 $$\begin{aligned} \mathbf {s}(y) = \underbrace{\int _\varOmega {\mathbf {K}(x,y)\mathrm{d}x}}_{(\mathrm{a})} = \underbrace{\left( \int _\varOmega {k_{ij}(x,y)\mathrm{d}x}\right) }_{(\mathrm{b})} = \underbrace{\left( s_{ij}(y)\right) }_{(\mathrm{c})} \end{aligned}$$

where (a) is integration over a matrix, each entry is integrated irrespective of the others, and the brackets around (b) and (c) denote matrices. An average over $\mathbf {s}(y)$ for all of the *y* values in $\varOmega$ yields the matrix form of the average dispersal success function,

29 $$\begin{aligned} \mathbf {S} = \frac{1}{|\varOmega |} \int _\varOmega {\mathbf {s}(y)\mathrm{d}y} = \frac{1}{|\varOmega |}\int _\varOmega {\int _\varOmega {\mathbf {K}(x,y)\mathrm{d}x}\mathrm{d}y}, \end{aligned}$$

where again the integration is over each entry in the matrix. $\mathbf {\widehat{S}}$ follows naturally from this,

30 $$\begin{aligned} \mathbf {\widehat{S}} = \frac{1}{|\varOmega |} \int _\varOmega {\frac{\mathbf {s}^2(y)}{\mathbf {S}}\mathrm{d}y}, \end{aligned}$$

where $\mathbf {s}^2(y)/\mathbf {S}$ should be interpreted element by element. Now, (4) extends to a spatially implicit matrix model,

31 $$\begin{aligned} \bar{\mathbf {N}}_{t+1} = [\mathbf {A}\circ \mathbf {R}(\bar{\mathbf {N}}_t)]\bar{\mathbf {N}}_t, \end{aligned}$$

where $\mathbf {A} = \mathbf {S}$ or $\mathbf {\widehat{S}}$. Note that for any sedentary stages whose entry in $\mathbf {K}(x,y)$ is the Dirac delta function, the corresponding entry in either $\mathbf {S}$ or $\mathbf {\widehat{S}}$ will be 1, as all individuals who start in the domain will stay in the domain, since they do not disperse at all.

#### Structured Population Example

We have chosen a stage-structured population with two stages for an illustrative example,

32 $$\begin{aligned} \mathbf {N}(x) = \left[ \begin{array}{cc} N_1(x) \\ N_2(x) \end{array} \right] . \end{aligned}$$

$N_1$ represents the dispersing population of juveniles and $N_2$ any individuals which have been recruited after settling. Only the second stage reproduces, and reproduction is density dependent, with the growth rate monotonically decreasing as a function of $N_2$ density, such as $r(1-N_2(x))$. We assume that individuals in stage $N_1$ either grow from stage $N_1$ to stage $N_2$ at rate $\alpha$ or they die. Individuals in stage $N_2$ have survivorship $\beta$. This results in the transition matrix

33 $$\begin{aligned} \mathbf {R}(\mathbf {N}(x)) = \left[ \begin{array}{cc} 0 &{}\quad r(1 - N_2(x)) \\ \alpha &{}\quad \beta \end{array}\right] . \end{aligned}$$

The resulting non-spatial model is the matrix population model of the form

34 $$\begin{aligned} \mathbf {N}_{t+1} = \mathbf {R}(\mathbf {N}_t)\mathbf {N}_t, \end{aligned}$$

for which the zero steady state is linearly unstable provided $r > (1-\beta )/\alpha$. If this were not the case, it would be futile to consider the critical domain size of the spatial model, as the population could not persist on a domain of any size. We assume that the population disperses only during reproduction (for our purposes, we will assume a Laplace dispersal kernel in $k_{12}$ and $\delta (x,y)$ elsewhere) and so

35 $$\begin{aligned} \mathbf {N}_{t+1}(x) = \int _{-L/2}^{L/2}{\left( \left[ \begin{array}{cc} \delta (x,y) &{}\quad k_{12} \\ \delta (x,y) &{}\quad \delta (x,y) \end{array}\right] \circ \mathbf {R}(\mathbf {N}(y))\right) \mathbf {N}_t(y)\mathrm{d}y}. \end{aligned}$$

**Fig. 7:**
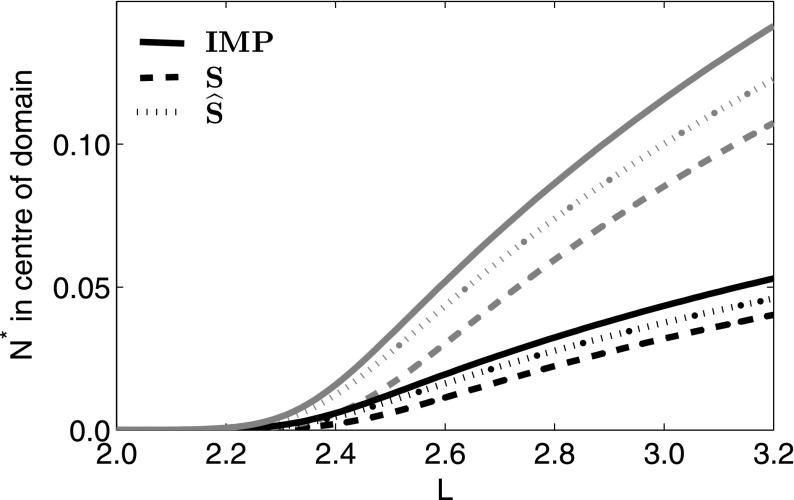
Comparison of the stable steady state of example (35) and the two approximations, *S* and $\widehat{S}$. *Black* represents those in class $N_2$ and *grey* represents those in the juvenile stage, $N_1$. Parameters are $r = 0.6$, $\alpha = 0.8$, $\beta = 0.7$, and *L* is scaled to the mean dispersal distance. Initial conditions are population density of 0.5 of both stages everywhere in the domain

**Fig. 8:**
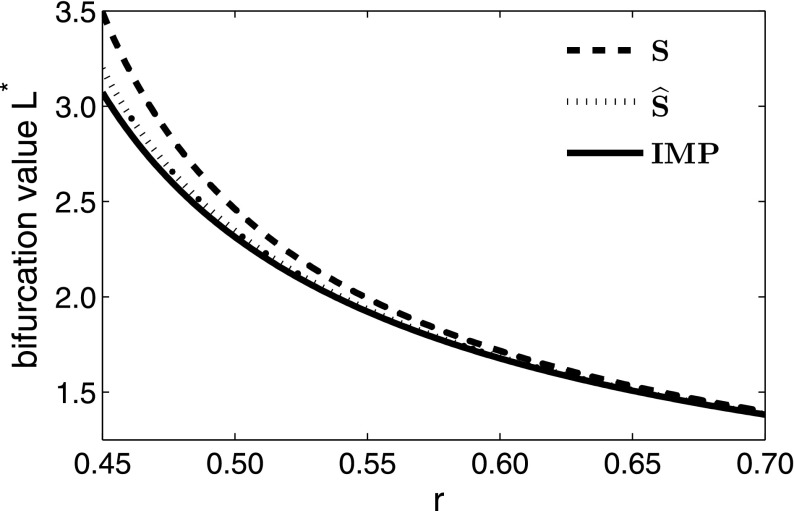
Comparison of the critical domain length as determined by the integrodifference matrix population model (IMP) and two approximations to (35) where $\alpha = 0.8$ and $\beta = 0.7$. Once again the modified dispersal success approximation remains closer to the IMP solution than the standard dispersal success function, especially for values of *r* close to the critical value, past which the population cannot persist on a domain of any size

From this, we obtain the critical domain size $L^*$,

36 $$\begin{aligned} L^* = (A + 2n\pi )\frac{b}{2a} \end{aligned}$$

where $A = \arctan {\left( \frac{4a(a^2-1)}{1-6a^2 + a^4}\right) } + \pi$, $a =\sqrt{\frac{r\alpha }{1-\beta } - 1}\,\,$ and $b = \sqrt{D/\alpha }\,\,$ (see Stage-Structured Population Theory section of Appendix 2 for details). As in the scalar case, for certain parameter regimes a bifurcation from the trivial steady state to a positive non-zero one is possible and dependent on both the demographic parameters and the domain size. Calculating *S* and $\widehat{S}$ as above for this example (35), we see again that $\widehat{S}$ outperforms *S* (Figs. 7, 8).

### Incorporating Alongshore Currents

An important component of our systems of interest may be advective forces. Many coastal or reef populations experience unidirectional drift either due to alongshore currents created by wind and waves, or larger ocean currents. Prevailing winds may cause seed dispersal to tend heavily in one direction. Unidirectional currents can create sink-source dynamics and extinction of upstream populations if insufficient recruitment occurs (Speirs and Gurney 2001).

Some have attempted to incorporate advection into the dispersal kernel by simply shifting the mode of the kernel downstream by some advective factor (Lockwood et al. 2002; Lutscher et al. 2010; Neubert et al. 1995). This is not biologically appropriate for many kernels, which are derived from the important assumption that not all larvae settle at the same time. We here direct the reader to the work of Lutscher et al. (2005) who derived modified dispersal kernels to be used in an integrodifferential equation approach to modelling organisms in river systems.

**Table 2:** Dispersal kernels from Table 1 subject to advection with effective drift rate *v*, stopping time $t_0$ or stopping rate $\alpha$, as derived in Lutscher et al. (2005)

*Dispersal kernels subject to advection*	
Shifted Gaussian	$k(x,y) = \frac{\exp {\left( \frac{-(x - vt_0 - y)^2}{4Dt_0}\right) }}{\sqrt{4\pi Dt_0}}$
Asymmetric Laplace	$$k(x,y) = {\left\{ \begin{array}{ll}A\exp {(a_1(x-y))}, &{} (x-y) \le 0 \\ A\exp {(a_2(x-y))}, &{} (x-y) > 0 \end{array}\right. }$$
Shifted Cauchy	$k(x,y) = \frac{t_0}{\rho \pi }\left[ \left( \frac{x - vt_0-y}{\rho }\right) ^2 + t_0^2\right] ^{-1}$
Asymmetric fat-tailed	$k(x,y) = \frac{\alpha }{(\mu ^2 + v^2)\pi } \mathfrak {R}\left( (\mu + vi)\hbox {E}_1\left( \frac{\alpha (v - \mu i)(y-x)}{\mu ^2 + v^2}\right) \exp \left( -\frac{\alpha (v - \mu i)(x-y)}{\mu ^2 + v^2}\right) \right)$

$A = a_1 a_2/(a_2 - a_1) = \alpha /\sqrt{v^2+4\alpha D}$, $a_{1,2} = v/2D \pm \sqrt{v^2/4D^2 + \alpha /D}$. Note that $\sigma = \sqrt{Dt_0}$ in Table 1

The resulting dispersal kernels can be found in Table 2, and we refer to Lutscher et al. (2005) for an overview of the derivation procedure. When the assumption is that the dispersers all settle at the same time $t_0$, advection simply shifts the kernel by $v t_0$, where *v* is the strength of advection. If the population settles at a constant rate $\alpha$, then the shape of the kernel changes to become asymmetrical. We here consider our modified average dispersal success function as well as that of Van Kirk and Lewis (1997) in the presence of advection.

#### Example with Advection

We again turn to the Laplace dispersal kernel and logistic growth for an illustrative example. Using the standard method, we find the critical domain size $L^*$ by differentiating the IDE twice with respect to *x* and applying the relevant boundary conditions (see Example in an Advective Environment section of Appendix 2 for details; Kot and Schaffer 1986; Lutscher et al. 2010, 2005; Pachepsky et al. 2005). The critical domain size for this example is

37 $$\begin{aligned} L^* = \frac{4\arctan {\left( \sqrt{\frac{4ra_1|a_2|}{(a_1-a_2)^2}-1}\right) ^{-1}}}{(a_1-a_2)\sqrt{\frac{4ra_1|a_2|}{(a_1-a_2)^2}-1}}, \end{aligned}$$

where $a_1$ and $a_2$ are as in Table 2. Note that this is a decreasing function in *r*, which we would expect, since the more fecund a species is, the smaller the proportion of individuals which need to be retained inside the domain for persistence. The critical domain size $L^* \rightarrow \infty$ as

$$\begin{aligned} r \rightarrow \frac{(a_1-a_2)^2}{4a_1|a_2|}, \end{aligned}$$

so the persistence threshold is

$$\begin{aligned} r^* = \frac{(a_1-a_2)^2}{4a_1|a_2|}. \end{aligned}$$

For any $r>r^*$, the population will persist on a domain of length $L > L^*$ (Lutscher et al. 2010). Lutscher et al. (2005) found that at low advective speeds, as in the case without advection, the critical domain size is most influenced by the mean dispersal distance rather than rare long-distance events. In the presence of increased advection, however, the critical domain size depends heavily on rare long-distance events, as it is the few rare individuals who disperse upstream which are able to prevent the population from being washed out of the patch entirely (Lutscher et al. 2005).

Intuitively, the critical domain size increases with advection as more individuals are lost from the downstream edge and a larger domain is required to retain enough individuals for each generation to persist. This continues until, for some critical advective speed, $v_p$, there is no finite domain large enough to allow for persistence (Lutscher et al. 2010). We investigate whether our approximations are still useful if we incorporate advection. For the asymmetric Laplace kernel, *s*(*y*) and *S* are

38 $$\begin{aligned} s(y) = \frac{A\, \left( \mathrm {e}^{\frac{{a_2}\, (L - 2\, y)}{2}} - 1\right) }{{a_2}} - \frac{A\, \left( \mathrm {e}^{\frac{{-a_1}\, (L + 2\, y)}{2}} - 1\right) }{{a_1}}, \end{aligned}$$

and

39 $$\begin{aligned} S = \frac{A\, \left( \mathrm {e}^{-a_1 L} + a_1 L - 1\right) }{L{a_1}^2} - \frac{A\, \left( a_2 L - \mathrm {e}^{a_2 L} + 1\right) }{L {a_2}^2} \end{aligned}$$

where *A*, $a_1$, and $a_2$ are as in Table 2 and *L* is scaled to the mean dispersal distance. When we start to look at the modified average dispersal success, though, we encounter a problem in our assumptions. The motivation behind the modified average dispersal success function comes from the idea that we can estimate the long-term behaviour of a population by the dispersal success function, *s*(*y*). It does not, however, make sense to assume this to be the shape of the population as we previously did, since now most of the dispersers which settle inside the domain will settle near the downstream edge while dispersal success will be highest for individuals near the upstream edge. To reconcile this with our approximation, we employ the redistribution function *r*(*x*) introduced in Lutscher and Lewis (2004) and defined as

40 $$\begin{aligned} r(y) = \int _\varOmega {k(y,x)\mathrm{d}x}. \end{aligned}$$

For the asymmetric Laplace kernel, this is

41 $$\begin{aligned} r(y) = \frac{A\, \left( \mathrm {e}^{{a_2}\, \left( \frac{L}{2} + y\right) } - 1\right) }{{a_2}} - \frac{A\, \left( \mathrm {e}^{{a_1}\, \left( y-\frac{L}{2}\right) } - 1\right) }{{a_1}}. \end{aligned}$$

The difference between *r*(*y*) and *s*(*y*) lies in the switch between *k*(*x*, *y*) and *k*(*y*, *x*). For symmetric kernels on an isotropic domain, $k(x,y) = k(y,x)$ and there is no need for a different measure of a population’s successful redistribution. However, in the event that dispersal depends explicitly on the starting location or when the kernel is not symmetric, *r*(*y*) provides a better estimate for the shape of the population distribution than *s*(*y*). We can think of *s*(*y*) as the probability that a dispersing individual which begins at *y* settles anywhere inside the domain by the end of a time step, while *r*(*y*) is the probability that a propagule which begins anywhere in the domain ends up at point *y* by the end of a time step. This captures the downstream tendencies of populations subject to advection better than *s*(*y*). If the kernel does not depend explicitly on space, the two curves are mirror images of each other reflected across the centre of the domain and so the average dispersal success is defined as

42 $$\begin{aligned} S = \frac{1}{|\varOmega |}\int _\varOmega {s(y)\mathrm{d}y} = \frac{1}{|\varOmega |}\int _\varOmega {r(y)\mathrm{d}y}. \end{aligned}$$

When attempting to approximate the population via the newly introduced modified average dispersal success function, we will multiply the average dispersal success function’s integrand by *r*(*y*) / *S* rather than *s*(*y*) / *S* as we previously did, in order to capture both the population distribution shifting downstream and the dispersal success of the individuals in that distribution, so that the modified dispersal success function subject to advection is

43 $$\begin{aligned} \widehat{S} = \int _{\varOmega }{\frac{r(y)}{S}s(y)\mathrm{d}y}. \end{aligned}$$

For the asymmetric Laplace kernel, this is

44 $$\begin{aligned} \widehat{S}= & {} \left[ \frac{1}{{a_1}{a_2}\left( {a_1} - {a_2}\right) \left( \mathrm {e}^{L{a_1}}\left( {{a_1}}^2\mathrm {e}^{L{a_2}} - {{a_1}}^2 - {{a_2}}^2 + L{a_1}{{a_2}}^2 - L{{a_1}}^2{a_2}\right) + {{a_2}}^2\right) }\right] \nonumber \\&\times \left[ - A \left( \big (2 {{a_1}}^4 + 2{{a_1}}^2{{a_2}}^2 - 4{{a_1}}^3{a_2} + L{{a_1}}^3{{a_2}}^2 - L {{a_1}}^4 {a_2} \big ) \mathrm {e}^{L \left( {a_1} + {a_2}\right) } \right. \right. \nonumber \\&\left. + \left( - 4 {a_1} {{a_2}} + 2 {{a_2}}^2 + 2{{a_1}}^2 - L{{a_1}}^2{{a_2}} - 2{{a_1}}^2\mathrm {e}^{L{a_2}} + L{a_1}{{a_2}}^2 \right) {{a_2}}^2 \right. \nonumber \\&+ \left( - 2 {{a_1}}^4 - 2 {{a_2}}^4 - 2{{a_1}}^2{{a_2}}^2 + 4{a_1}{{a_2}}^3 + 4 {{a_1}}^3 {a_2} + L {a_1} {{a_2}}^4 - L{{a_1}}^4{a_2}\right. \nonumber \\&\left. \left. \left. - 3 L {{a_1}}^2 {{a_2}}^3 + 3 L{{a_1}}^3 {{a_2}}^2 \right) \mathrm {e}^{L {a_1}}\right) \right] . \end{aligned}$$

**Fig. 9:**
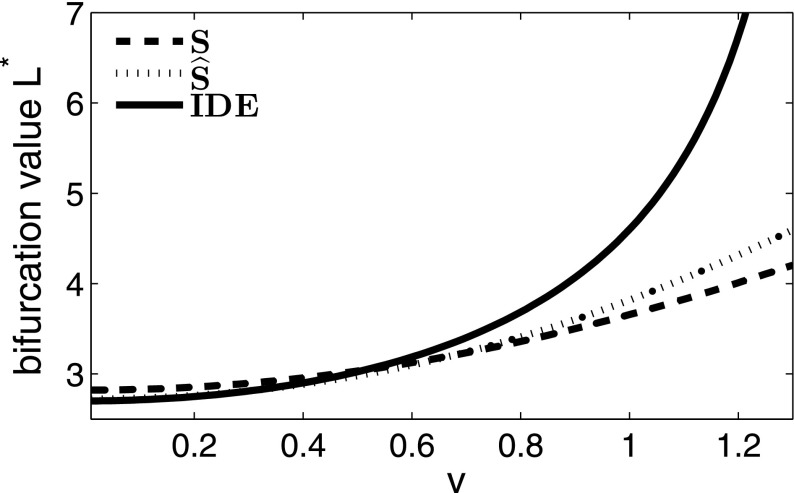
Critical domain size of an IDE with an asymmetric Laplace dispersal kernel and logistic growth with $r = 1.5$, as compared with the two approximations. As *v* increases towards its critical value, where the population cannot persist on a domain of any size, the approximations diverge from the IDE

However, in spite of these changes to $\widehat{S}$, the IDE is still far more sensitive to advection than either the *S* or $\widehat{S}$ approximations. As advection strength increases (Fig. 9), the dispersal success and redistribution functions are no longer a close approximation to the shape of the equilibrium solution, and this is only worsened with increasing advection. In Fig. 9, for small values of advection, the approximations predict critical domain sizes close to that of the IDE, but as advection increases, the approximations grow increasingly further away from the IDE value until the advection value reaches $V_p$, where the population cannot persist on a domain of any size as all individuals are swept downstream.

This discrepancy is due to the inability of the spatially implicit approximation to capture the effects of washout. In the spatially implicit approximation, the population is swept downstream but so long as the domain is large enough, it can remain within the patch (note that there is no critical advection value for the approximations for this reason). The IDE, however, captures the fact that the population will tend to zero upstream, as the population is washed downstream.

## Discussion

While IDEs provide us with a way to model a variety of dispersal patterns and growth functions, they are seldom analytically tractable and the critical domain size can often only be obtained via methods involving numerical integration. This makes it difficult to gain insights into the effects of various parameters or the properties of the growth functions or dispersal kernels. Approximations to an IDE framework are thus useful for those wishing to compare the effects of different dispersal patterns and demographic rates.

The standard approximation of dispersal success has typically been that of Van Kirk and Lewis (1997). Their approximation assumes that the population is evenly distributed through the domain, but this is not generally the case. For typical populations with unimodal symmetric dispersal kernels, areas near the hostile boundaries have a smaller domain from which they may recruit individuals. Thus, there will tend to be more individuals in the centre of a patch, away from the boundaries. One way to approximate the distribution of individuals inside the domain is by using the dispersal success function at each point in the domain (Van Kirk and Lewis 1997). Recall that for symmetric kernels, the dispersal success function *s*(*y*) is the same as the redistribution function *r*(*y*), the probability that an individual who begins anywhere in the domain settles at location *y*. Thus, locations with higher dispersal success also have more individuals settling there from elsewhere in the domain and can sustain larger population sizes. By accounting for this non-homogeneous population distribution, the new approximation is able to outperform the standard one which assumes that the population is evenly distributed. Using this to provide a weighted average of the dispersal success across the domain leads to a better approximation to the proportion of individuals lost due to dispersal outside the domain and thus a better approximation of population growth.

The two approximations vary most, both from each other and from the value of the IDE, for parameter regimes close to critical demographic values. Whether a population of interest has parameters in these regions will be species and environment specific, but since the new approximation provides a closer approximation to the critical domain size even in these regions, it should be used in place of the average dispersal success function.

Our modified dispersal success function results in a spatially implicit approximation to an IDE with a domain of a single large patch, an idealized network of infinitely many evenly sized and spaced patches, and stage- or size-structured populations. We also considered populations in areas subject to advection, but here our approximation did not respond to changes in advection speed in the same way that the IDE did, resulting in inaccurate predictions of the critical domain size. Looking at where both the Van Kirk and Lewis (1997) approximation and our new approximation fail sheds some light on the dynamics of populations subject to advection. The explicit spatial dependence of an IDE allows it to capture the effects of “wash-out,” where the population is swept to the downstream edge of the domain before being swept into hostile habitat. Our spatially implicit averaging techniques cannot account for the compounding effects of advection. In this case, the new approximation outperforms that of Van Kirk and Lewis (1997) for certain parameter regimes when advection is not very strong, but these approximations should only be used very cautiously if considering populations subject to strong advection.

By examining these approximations, we gain general insight about IDEs. Since being able to approximate the proportion of individuals which successfully settle within the domain results in a critical domain size close to that of the original IDE, we can infer that this is a significant aspect of dispersal which drives the bifurcation behaviour of an IDE. Even if a suitable mechanistic kernel is not available, if the proportion *A* of individuals locally retained can be determined empirically, the resulting necessary domain size can be established using (4). Furthermore, if we can predict the proportion of larvae lost outside a given domain in more dimensions, (4) incorporates this easily, as opposed to an IDE framework, which would then require multidimensional integration.

We have found that the modified dispersal success approximation provides close estimates to the behaviour of a population modelled using an IDE framework. This new approximation allows us to approximate the domain size necessary for population persistence, based on, and closely mimicking, more complicated IDEs. This framework is suitable for many species of interest (e.g. many marine species and nearly all plant species), and the approximation we have presented allows for insights into population persistence in a variety of environments and for populations with different dispersal or reproductive behaviour.

## Appendix 1: Proof that $\widehat{S} \ge S$

Assume *s*(*y*) is piecewise continuous (this holds for the dispersal kernels considered in this paper). WLOG, let the domain of interest be $\varOmega = [0,1]$ so $|\varOmega | = 1$. For notational ease, we suppose $s(y) < S$ on $\varOmega _1 \subset \varOmega$ and $s(y) \ge S$ on $\varOmega _2 \subseteq \varOmega$. On $\varOmega _1$, we write $s(y) = \alpha (y)S$ with $\alpha (y) \in [0,1), \, \forall y \in \varOmega _1$ and on $\varOmega _2$, we write $s(y) = \beta (y)S$ for $\beta (y) \ge 1,\, \forall y \in \varOmega _2$. Then,

45 $$\begin{aligned} \begin{aligned} S&= \int _\varOmega {s(y)\mathrm{d}y}\\&= \int _{\varOmega _1}{s(y)\mathrm{d}y} + \int _{\varOmega _2}{s(y)\mathrm{d}y}\\&= \int _{\varOmega _1}{\alpha (y)S \mathrm{d}y} + \int _{\varOmega _2}{\beta (y)S \mathrm{d}y}\\&= S\left( \int _{\varOmega _1}{\alpha (y)\mathrm{d}y} + \int _{\varOmega _2}{\beta (y)\mathrm{d}y} \right) . \end{aligned} \end{aligned}$$

Dividing through by *S* and letting $\alpha (y) = 1 - \hat{\alpha }(y)$ and $\beta (y) = 1 + \hat{\beta }(y)$ for $\hat{\alpha } > 0$, $\hat{\beta } \ge 0$ yields

46 $$\begin{aligned} \begin{aligned} 1&= \int _{\varOmega _1}{1-\hat{\alpha }(y)\mathrm{d}y} + \int _{\varOmega _2}{1 + \hat{\beta }(y)\mathrm{d}y} \\&= \int _{\varOmega }{1} - \int _{\varOmega _1}{\hat{\alpha }(y)\mathrm{d}y} + \int _{\varOmega _2}{\hat{\beta }(y)\mathrm{d}y}\\ \Rightarrow 0&= - \int _{\varOmega _1}{\hat{\alpha }(y)\mathrm{d}y} + \int _{\varOmega _2}{\hat{\beta }(y)\mathrm{d}y}. \end{aligned} \end{aligned}$$

In a similar fashion,

47 $$\begin{aligned} \widehat{S}= & {} \int _\varOmega {\frac{s(y)}{S}s(y)\mathrm{d}y}\nonumber \\= & {} \int _{\varOmega _1}{\frac{s(y)}{S}s(y)\mathrm{d}y} + \int _{\varOmega _2}{\frac{s(y)}{S}s(y)\mathrm{d}y} \nonumber \\= & {} \int _{\varOmega _1}{\frac{\alpha (y)S}{S}\alpha (y)Sdy} + \int _{\varOmega _2}{\frac{\beta (y)S}{S}\beta (y)Sdy} \nonumber \\= & {} S\left( \int _{\varOmega _1}{\alpha ^2(y)\mathrm{d}y} + \int _{\varOmega _2}{\beta ^2(y)\mathrm{d}y} \right) \nonumber \\= & {} S\left( \int _{\varOmega _1}{(1-\hat{\alpha }(y))^2\mathrm{d}y} + \int _{\varOmega _2}{(1+\hat{\beta }(y))^2\mathrm{d}y} \right) \nonumber \\= & {} S\left( \int _{\varOmega }{1 \mathrm{d}y} + \int _{\varOmega _1}{\hat{\alpha }^2(y)\mathrm{d}y} + \int _{\varOmega _2}{\hat{\beta }^2(y)\mathrm{d}y} - 2\int _{\varOmega _1}{\hat{\alpha }(y)\mathrm{d}y} + 2\int _{\varOmega _2}{\hat{\beta }(y)\mathrm{d}y}\right) \quad \nonumber \\= & {} S\left( 1 + \int _{\varOmega _1}{\hat{\alpha }^2(y)\mathrm{d}y} + \int _{\varOmega _2}{\hat{\beta }^2(y)\mathrm{d}y}\right) \hbox {by (46)}\nonumber \\\ge & {} S.\quad \end{aligned}$$

$$\square $$

## Appendix 2: Theory and Details from the Examples

### Standard IDE Theory

Here, we remind the reader of the key results and methods of Kot and Schaffer (1986) and provide a simple example which we use in comparison with our approximations. Assuming that the growth function is the same everywhere, independent of location, i.e. $f(N_t(y);y) = f(N_t(y))$, Kot and Schaffer examined the fixed points of a scalar, nonlinear IDE on a closed domain $\varOmega = [-L/2, L/2]$. Following the standard method for discrete models (see Kot and Schaffer 1986), they attempted to find $N^*(x)$ satisfying

48 $$\begin{aligned} N^*(x) = \int _{-L/2}^{L/2}{k(x,y)f(N^*(y))\mathrm{d}y}. \end{aligned}$$

The similarities between difference equations with no spatial component and the IDEs considered can be seen by introducing a nonlinear operator $\mathcal {F}$ so that

49 $$\begin{aligned} N_{t+1}(x) = \mathcal {F}N_t(x) = \int _{-L/2}^{L/2}{k(x,y)f(N_t(y))\mathrm{d}y} \end{aligned}$$

and Eq. (48) can be rewritten as $N^*(x) = \mathcal {F}N^*(x)$ (Kot and Schaffer 1986). For our purposes, *f*(*N*(*x*)) can typically be expressed as $f(N(x)) = N(x)g(N(x))$ [see (1)], where *g*(*N*(*x*)) is a well-behaved function describing the per capita growth rate. We thus assume $N^*(x) = 0$ is always a trivial solution. Obtaining analytic expressions for any non-trivial equilibria will depend on the kernel and growth function employed. To determine the linear stability of an equilibrium, Kot and Schaffer (1986) considered a small perturbation $\xi _t(x)$ away from the equilibrium, so that $N_t(x) = N^*(x) + \xi _t(x)$. For sufficiently small $\xi _t(x)$, this is equivalent to studying the linearization of the IDE around the steady state. Considering the nonlinear operator $\mathcal {F}$, this is its Fréchet differential, where

50 $$\begin{aligned} \xi _{t+1}(x) = (\mathcal {F}'N^*(x))(\xi _t(x)) = \int _{-L/2}^{L/2}{k(x,y)\left[ \frac{\partial f(N(y))}{\partial N(y)}\bigg |_{N^*}\right] \xi _t(y)\mathrm{d}y}. \end{aligned}$$

The asymptotic stability of the equilibrium $N^*(x)$ is thus determined by the Fréchet derivative $\mathcal {F}'N^*(x)$. For finite limits of integration and continuous kernels (which are likely, given the question of critical domain size and realistic dispersal behaviour within the population), this Fréchet derivative is a compact operator (Kot and Schaffer 1986). As such, its spectrum consists of, at most, the point zero and a countable number of non-zero eigenvalues (Conway 1990; Kot and Schaffer 1986). The stability of the equilibrium is determined by examining the magnitude of these eigenvalues. Kot and Schaffer (1986) assumed that $\xi _t(x)$ is of the form

$$\begin{aligned} \xi _t(x) = \lambda ^t\mu (x), \end{aligned}$$

where $\lambda$ represents the eigenvalues and $\mu (x)$ the non-zero eigenvectors. They arrived at this form by analogy with the separation of variables method commonly used with linear partial differential equations. Assuming this form for $\xi _t(x)$ leads, along with Eq. (50), to

51 $$\begin{aligned} \lambda \mu (x) = (\mathcal {F}'N^*)\mu (x) = \int _{-L/2}^{L/2}{k(x,y)\left[ \frac{\partial f(N(y))}{\partial N(y)}\bigg |_{N^*}\right] \mu (y)\mathrm{d}y}. \end{aligned}$$

If all eigenvalues $\lambda$ lie within the unit circle of the complex plane (i.e. $|\lambda | <1$), then $N^*(x)$ is locally asymptotically stable. Kot and Schaffer (1986) have shown that, given certain conditions of non-negativity and symmetry in the kernel, as well as non-negativity of $\mathrm{d}f(N^*(x))/\mathrm{d}N$, there exists a positive non-zero dominant eigenvalue, so stability of the trivial steady state will be lost when $\lambda = 1$. Furthermore, Hardin et al. (1990) have shown that for monotonically increasing and bounded growth functions (e.g. Beverton–Holt, Table 1), the trivial equilibrium solution is globally stable when its dominant eigenvalue is less than 1 and the non-trivial equilibrium solution is globally stable otherwise (Hardin et al. 1990).

#### Standard IDE Example

We consider our carefully chosen example of Sect. 3.1, following the methods of Kot and Schaffer (1986). We chose the Laplace dispersal kernel (Table 1) as it displays convenient properties allowing for analytic tractability, as pointed out in Kot and Schaffer (1986). Assuming logistic growth (Table 1), this results in the following IDE

52 $$\begin{aligned} N_{t+1}(x) = \sqrt{\frac{\alpha }{4D}}\int _{-L/2}^{L/2}{\exp {\left( -|x-y|\sqrt{\frac{\alpha }{D}}\right) }f(N_t(y))\mathrm{d}y}, \end{aligned}$$

where $f(N_t(y)) = r\, N_t(y)\left( 1 - N_t(y)\right)$. Any fixed points are the solutions to

53 $$\begin{aligned} N^*(x) = \sqrt{\frac{\alpha }{4D}}\int _{-L/2}^{L/2}{\exp {\left( -|x-y|\sqrt{\frac{\alpha }{D}}\right) }f(N^*(y))\mathrm{d}y}. \end{aligned}$$

Omitting the $*$ for notational convenience, we continue to follow Kot and Schaffer (1986) and differentiate the above with respect to *x*, which yields

54 $$\begin{aligned} \begin{aligned} N'(x) =&\frac{\alpha }{2D}\Bigl [-\int _{-L/2}^{x}{\exp {\left( (y-x)\sqrt{\frac{\alpha }{D}}\right) }f(N(y))\mathrm{d}y} \\&+\,\int _{x}^{L/2}{\exp {\left( (x-y)\sqrt{\frac{\alpha }{D}}\right) }f(N(y))\mathrm{d}y}\Bigr ]. \end{aligned} \end{aligned}$$

Differentiating once more yields

55

and simplifying results in

56a $$\begin{aligned} N''(x) + \frac{\alpha }{D}[f(N(x)) - N(x)] = 0. \end{aligned}$$

Following Kot and Schaffer (1986), we look at $x = \pm L/2$ in (54) to obtain the relevant boundary conditions

56b $$\begin{aligned} N'(x)= & {} \sqrt{\frac{\alpha }{D}}N(x) \quad \hbox {at } x = -L/2, \nonumber \\ N'(x)= & {} -\sqrt{\frac{\alpha }{D}}N(x) \quad \hbox {at } x = L/2. \end{aligned}$$

We can clearly see that $N^*(x) = 0$ is a solution for any growth functions of the form $f(N(x)) = N(x)g(N(x))$. Substituting the logistic growth equation $f(N(x)) = r\, N(x)\left( 1 - N(x)\right)$, we obtain

57 $$\begin{aligned} N''(x) + N(x)\left( \frac{\alpha }{D}\right) [r\,\left( 1 - N(x)\right) - 1] = 0, \end{aligned}$$

with the same boundary conditions as above. $N^*(x) = 0$ is clearly a solution of (57), and we now look for any non-trivial equilibrium solutions. Following the method set out by Kot and Schaffer (1986), the most illuminating way to proceed is to turn (57) into a system of two ODEs by setting $u(x) = N(x)$ and $v(x) = N'(x)$. This results in

58a $$\begin{aligned} u'(x)&= v(x), \end{aligned}$$

58b $$\begin{aligned} v'(x)&= -u(x)\left( \frac{\alpha }{D}\right) [r\,\left( 1 - u(x)\right) - 1], \end{aligned}$$

with boundary conditions

58c $$\begin{aligned} \begin{array}{lcl} v(x) = \sqrt{\frac{\alpha }{D}}u(x) &{}\quad \hbox {at } &{}x = -L/2, \\ v(x) = -\sqrt{\frac{\alpha }{D}}u(x) &{}\quad \hbox {at } &{}x = L/2. \end{array} \end{aligned}$$

There are two equilibrium solutions $(u^*,v^*)$ at (0, 0) and (1, 0) to the non-spatial system,

59 $$\begin{aligned} u'&= v, \end{aligned}$$

60 $$\begin{aligned} v'&= -u\left( \frac{\alpha }{D}\right) [r\,\left( 1 - u\right) - 1]. \end{aligned}$$

Solutions corresponding to our boundary conditions are orbits in the phase space beginning at $v = u\sqrt{\alpha /D}$ at $x = -L/2$ and ending on $v = -u\sqrt{\alpha /D}$ at $x = L/2$ (Kot and Schaffer 1986).

There is a domain threshold size, which we will denote as $L^*$, at which reproduction is exactly sufficient to balance the cost of individuals dispersing to the hostile environment outside the domain. The critical domain size $L^*$ is the bifurcation value for *L* at which stability switches from the trivial steady state $N^* = 0$ to a non-trivial steady state. To find $L^*$, we look at where the trivial solution loses stability and recall that the trivial solution is locally asymptotically stable if all of the eigenvalues $\lambda$ of (51) lie within the unit circle of the complex plane. For the chosen kernel, which is continuous, symmetric, and non-negative, all eigenvalues are real and positive, and stability will be lost through $\lambda = 1$ (Kot and Schaffer 1986). For the Laplace kernel and logistic growth function chosen in this example, (51) becomes

61 $$\begin{aligned} \lambda \mu (x) = r \sqrt{\frac{\alpha }{4D}}\int _{-L/2}^{L/2}{\exp {(-|x-y| \sqrt{\alpha /D})}\mu (y)\mathrm{d}y}. \end{aligned}$$

Differentiating twice, we find

62a $$\begin{aligned} \mu ''(x) + \left( \frac{\alpha }{D}\right) \frac{r - \lambda }{\lambda }\mu (x) = 0, \end{aligned}$$

with boundary conditions

62b $$\begin{aligned} \begin{array}{lcl} \mu '(x) = \mu (x)\sqrt{\frac{\alpha }{D}} \quad &{}\hbox {at}\quad &{}x = -L/2, \\ \mu '(x) = -\mu (x)\sqrt{\frac{\alpha }{D}} \quad &{}\hbox {at} \quad &{}x = L/2. \end{array} \end{aligned}$$

Following Kot and Schaffer (1986), we let

63 $$\begin{aligned} \gamma ^2 = \frac{r - \lambda }{\lambda }, \end{aligned}$$

so we have

64 $$\begin{aligned} \mu ''(x) + \left( \gamma \sqrt{\frac{\alpha }{D}}\right) ^2\mu (x) = 0, \end{aligned}$$

and we can assume a solution of the form

$$\begin{aligned} \mu (x) = A\cos {\left( \gamma \sqrt{\frac{\alpha }{D}}x\right) } + B\sin {\left( \gamma \sqrt{\frac{\alpha }{D}}x\right) }, \end{aligned}$$

for some constants *A* and *B*. In order to satisfy the boundary conditions,

65 $$\begin{aligned} \begin{aligned} \mu '(-L/2)&= -A\gamma \sqrt{\frac{\alpha }{D}}\sin {\left( -\gamma \sqrt{\frac{\alpha }{D}}\frac{L}{2}\right) } + B\gamma \sqrt{\frac{\alpha }{D}}\cos {\left( -\gamma \sqrt{\alpha }{D}\frac{L}{2}\right) } \\&= A\sqrt{\frac{\alpha }{D}}\cos {\left( -\gamma \sqrt{\alpha }{D}\frac{L}{2}\right) } + B\sqrt{\frac{\alpha }{D}}\sin {\left( -\gamma \sqrt{\frac{\alpha }{D}}\frac{L}{2}\right) } \\&= \sqrt{\frac{\alpha }{D}}\mu (-L/2) \end{aligned} \end{aligned}$$

and

66 $$\begin{aligned} \begin{aligned} \mu '(L/2)&= -A\gamma \sqrt{\frac{\alpha }{D}}\sin {\left( \gamma \sqrt{\frac{\alpha }{D}}\frac{L}{2}\right) } + B\gamma \sqrt{\frac{\alpha }{D}}\cos {\left( \gamma \sqrt{\frac{\alpha }{D}}\frac{L}{2}\right) } \\&= -A\sqrt{\frac{\alpha }{D}}\cos {\left( \gamma \sqrt{\frac{\alpha }{D}}\frac{L}{2}\right) } - B\sqrt{\frac{\alpha }{D}}\sin {\left( \gamma \sqrt{\frac{\alpha }{D}}\frac{L}{2}\right) }\\&= -\sqrt{\frac{\alpha }{D}}\mu (L/2). \end{aligned} \end{aligned}$$

Rearranging yields two equations in two variables,

67 $$\begin{aligned}&A\left[ 1-\gamma \tan {\left( \gamma \sqrt{\frac{\alpha }{D}}\frac{L}{2}\right) }\right] - B\left[ \tan {\left( \gamma \sqrt{\frac{\alpha }{D}}\frac{L}{2}\right) }+\gamma \right] = 0\nonumber \\&A\left[ 1-\gamma \tan {\left( \gamma \sqrt{\frac{\alpha }{D}}\frac{L}{2}\right) }\right] + B\left[ \tan {\left( \gamma \sqrt{\frac{\alpha }{D}}\frac{L}{2}\right) }+\gamma \right] = 0, \end{aligned}$$

and so the eigenvalues $\lambda$ (recall $\gamma = \gamma (\lambda )$) will satisfy either transcendental equation

68 $$\begin{aligned} \tan {\left( \frac{\gamma L}{2}\sqrt{\frac{\alpha }{D}}\right) } = \pm \frac{1}{\gamma }. \end{aligned}$$

There are an infinite number of roots to these equations, hence an infinite number of eigenvalues. When the eigenvalues associated with $\gamma$ reach the unit circle at $\lambda = 1$ (i.e. when the stability of the trivial solution is lost), $\gamma = \sqrt{e^r -1}$ and the critical threshold $L^*$ must satisfy

69 $$\begin{aligned} \tan {\left( \frac{\sqrt{r-1}\,L^*}{2}\sqrt{\frac{\alpha }{D}}\right) } = \pm \frac{1}{\sqrt{r-1}}. \end{aligned}$$

Since the equation with the negative sign results in a negative value of $L^*$, which is not relevant to our present problem, the critical domain size for this example is

70 $$\begin{aligned} L^* = \frac{2}{\sqrt{r-1}}\sqrt{\frac{D}{\alpha }}\tan ^{-1}{\left( \frac{1}{\sqrt{r-1}}\right) }, \end{aligned}$$

as in (11).

### Example on a Network of Patches

We here work through the example of Sect. 4.1.1 on a network of patches for a population with a Laplace dispersal kernel and growth function *f*(*N*(*x*); *x*) of the form $$f(N(x);x) = N(x)g(N(x);x)$$ where $$g(\cdot ;x)$$ is periodic in *x* with period *L* and as described in the text, $$ f_2 = 0$$ represents a completely hostile landscape. We here follow the method outlined in Van Kirk and Lewis (1997) (see their work for a more thorough description of the framework). They begin by non-dimensionalizing the problem, dividing the spatial variables by the period *L*,

71 $$\begin{aligned} \tilde{x} = \frac{x}{L}\quad \hbox {and}\quad \tilde{y} = \frac{y}{L}. \end{aligned}$$

This results in the following IDE

72 $$\begin{aligned} N_{t+1}(L\tilde{x}) = \int _{-\infty }^{\infty }{\frac{L}{2b} \exp {\left( -\frac{L}{b}|\tilde{x}-\tilde{y}|\right) f(N_t(L\tilde{y});L\tilde{y})\,\mathrm{d}\tilde{y}},} \end{aligned}$$

where $b = \sqrt{D/\alpha }$. If we then let $\hat{L} = L/b$ (i.e. it is the measure of the effective spatial scale of habitat fragmentation, the period length divided by the mean dispersal distance), and $\tilde{N}_t(y) = N_t(L\tilde{y})$ we obtain the dimensionless IDE (dropping the tildes and hats for convenience)

73 $$\begin{aligned} N_{t+1}(x) = \int _{-\infty }^{\infty }{\frac{L}{2}\exp {(-L|x-y|)}f(N_t(y);y)\,\mathrm{d}y}, \end{aligned}$$

with equilibrium solutions satisfying

74 $$\begin{aligned} N^*(x) = \int _{-\infty }^{\infty }{\frac{L}{2}\exp {(-L|x-y|)}f(N^*(y);y)\,\mathrm{d}y}. \end{aligned}$$

Differentiating this twice with respect to *x* (and dropping the $*$ for convenience) yields the second-order differential equation

75 $$\begin{aligned} -\frac{1}{L^2}N''(x) + N(x) - N(x)g(N(x);x) = 0. \end{aligned}$$

Van Kirk and Lewis (1997) observed that $g(\cdot ;x)$ and its first derivative now have period 1, so the boundary conditions are $N(0) = N(1)$ and $N'(0) = N'(1)$. If we make a small perturbation around the zero steady state and assume that perturbation to be of the form $\xi _t(x) = \lambda ^t \mu (x)$, where $\lambda$ represents the eigenvalues and $\mu (x)$ the non-zero eigenvectors, we obtain the eigenvalue problem

76 $$\begin{aligned} -\frac{1}{L^2}\mu _{xx}(x) + \mu (x) = \frac{1}{\lambda }g(0;x)\mu (x), \end{aligned}$$

again with boundary conditions $\mu (0) = \mu (1)$ and $\mu '(0) = \mu '(1)$. Van Kirk and Lewis (1997) have shown that for operators like the one employed here, a single positive dominant eigenvalue $\lambda _1$ exists and corresponds to a positive eigenfunction $\mu _1(x)$. Now we take the periodic growth function *g*(0; *x*) found in Sect. 4.1.1, with alternating patches of “bad” and “good” habitat, where $g(0;x) = 0$ in the bad patches (the most extreme, conservative case) and $g(0;x) = r$ in the good patches. We let *R* be the fraction of domain in a good patch, and this results in a growth function of the form

77 $$\begin{aligned} g(0;x) = {\left\{ \begin{array}{ll} 0,&{}0<x<1-R\\ r,&{}1-R\le x \le 1.\end{array}\right. } \end{aligned}$$

The eigenvalue problem (76) is now given by

78 $$\begin{aligned} \mu _{xx} = {\left\{ \begin{array}{ll}L^2\mu , &{}\hbox {bad patches}, \\ -L^2(\frac{1}{\lambda _1}r-1)\mu , &{}\hbox {good patches}, \end{array}\right. } \end{aligned}$$

with the same boundary conditions as before. Because the IDE and its first derivative are continuous with respect to *x*, the eigenfunction $\mu (x)$ and its first derivative must be continuous at the discontinuity $x = 1-R$ of *g*(0; *x*) (Van Kirk and Lewis 1997). We search for a relationship between the length of the period (now a single variable *L* scaled to the mean dispersal distance) and the width of the good patches as a fraction *R* of *L*. We are interested in this relationship exactly at the bifurcation value $\lambda _1 = 1$, where the zero steady state loses stability. Setting $\lambda _1 = 1$ and substituting $G^2 = r - 1$, we have eigenfunctions of the form

79 $$\begin{aligned} \mu (x) = {\left\{ \begin{array}{ll} c_1 \exp {(Lx)} + c_2 \exp {(-Lx)}, &{} x \in (0, 1-R)\\ c_3 \sin {(GLx)} + c_4 \cos {(GLx)}, &{} x \in [1-R,1]. \end{array}\right. } \end{aligned}$$

Taking (79), we apply both the boundary conditions and the continuity of the eigenfunction and its first derivative at $x = 1-R$ to obtain

$$\begin{aligned} \mu (0)&= \mu (1)\\ \mu '(0)&= \mu '(1)\\ \mu (1-R)_-&= \mu (1-R)_+\\ \mu '(1-R)_-&= \mu '(1-R)_+ \end{aligned}$$

where $-$ denotes the limit from below and $+$ the limit from above. From this, we obtain four equations in four variables,

80 $$\begin{aligned} \begin{aligned} c_1 + c_2&= c_3 \sin {(\textit{LG})} + c_4 \cos (\textit{LG}) \\ c_1L - c_2L&= c_3 L G \cos (\textit{LG}) - c_4 L G \sin {(\textit{LG})}\\ c_1 \exp {(L(1-R))} + c_2 \exp {(-L(1-R))}&= c_3 \sin {(\textit{LG}(1-R))} + c_4 \cos {(\textit{LG}(1-R))}\\ c_1 L \exp {(L(1-R))} - c_2 L \exp {(-L(1-R))}&= c_3 L G \cos {(\textit{LG}(1-R))} \\&\quad \quad - c_4 \textit{LG}\sin {(\textit{LG}(1-R))}. \end{aligned} \end{aligned}$$

This can be written as the matrix equation

81 $$\begin{aligned} \mathbf {A}\mathbf {c} = \mathbf {0} \end{aligned}$$

where $\mathbf {A}$ is the augmented matrix

82 $$\begin{aligned} A \!=\! \left[ \begin{array}{cccc} 1 &{}\quad \! 1 &{}\quad \! -\sin {(\textit{LG})} &{}\quad \! -\cos {(\textit{LG})}\\ 1 &{}\quad \! -1 &{}\quad \! -G \cos {(\textit{LG})} &{}\quad \! G\sin {(\textit{LG})}\\ \exp {(L(1-R))} &{}\quad \! \exp {(-L(1-R))} &{}\quad \! -\sin {(\textit{LG}(1-R))} &{}\quad \! -\cos {(\textit{LG}(1-R))}\\ \exp {(L(1-R))} &{}\quad \! -\exp {(-L(1-R))} &{}\quad \! -G\cos {(\textit{LG}(1-R))} &{}\quad \! G\sin {(\textit{LG}(1-R))} \end{array} \right] , \end{aligned}$$

$\mathbf {c}$ is the column vector $[c_1, \ldots , c_4]^\mathrm{T}$ and $\mathbf {0}$ is the zero column vector. In order for there to exist a non-trivial solution to this system of equations, the relationship between the scaled periodicity *L* and the fraction of good habitat *R* must satisfy $\det {(\mathbf {A})} = 0$,

83 $$\begin{aligned} \begin{aligned} \det {(\mathbf {A})}&= 4G - \exp {(L - \textit{LR})}\sin {(\textit{GLR})} + \exp {(\textit{LR} - L)}\sin {(\textit{GLR})} \\&\quad - G^2\exp {(\textit{LR} - L)}\sin {(\textit{GLR})} - 2G\exp {(L - \textit{LR})}\cos {(\textit{GLR})} \\&\quad - 2G\exp {(\textit{LR} - L)}\cos {(\textit{GLR})} + G^2\exp {(L - \textit{LR})}\sin {(\textit{GLR})} = 0, \end{aligned} \end{aligned}$$

as in (21).

### Stage-Structured Population Theory

As with the basic IDE, we rewrite Eq. (27) as

84 $$\begin{aligned} \mathbf {N}_{t+1}(x) = \mathcal {F}(\mathbf {N}_t(x)) = \int _{\varOmega }[\mathbf {K}(x,y)\circ \mathbf {R}(\mathbf {N}_t(y);y)]\mathbf {N}_t(y)\mathrm{d}y \end{aligned}$$

where $\circ$ represents element-by-element matrix multiplication. We look for parameter values where the stable steady state solution bifurcates from the zero solution, which will be determined by looking at the leading eigenvalue of the nonlinear integral operator $\mathcal {F}$ in (84). We will here draw from the results on the existence and uniqueness of solutions of Lutscher and Lewis (2004). They have shown that under certain assumptions of compactness and differentiability, the nonlinear integral operator is positive, completely continuous, and strongly Fréchet differentiable at $\mathbf {N} = 0$ as given by

85 $$\begin{aligned} \mathcal {F'}(0)\pmb {\phi }(x) = \int _\varOmega {[\mathbf {K}(x,y)\circ \mathbf {R}(0,y)]\pmb {\phi }(y)\mathrm{d}y}. \end{aligned}$$

Given certain conditions on the primitivity of $\mathbf {R}(0,y)$, they have established the existence of a unique positive eigenvalue of the linear operator $\mathcal {F'}(0)$ with a corresponding positive eigenfunction. They obtain the superpositivity of $\mathcal {F}'(0)$ under further assumptions and so the dominant positive eigenvalue will determine the linear stability of the zero steady state. The stability of zero will be lost as the dominant eigenvalue, dependent on both the growth parameters and the domain length, passes through $\lambda = 1$. In the event that only juvenile individuals disperse, the dispersal matrix takes the form

86 $$\begin{aligned} \mathbf {K}(x,y) = \left[ \begin{array}{cccc} k_1(x,y) &{}\quad k_2(x,y) &{}\quad \ldots &{}\quad k_m(x,y) \\ \delta (x,y) &{}\quad \delta (x,y) &{}\quad \ldots &{}\quad \delta (x,y)\\ \vdots &{}\quad \vdots &{}\quad \ddots &{}\quad \vdots \\ \delta (x,y) &{}\quad \delta (x,y) &{}\quad \ldots &{}\quad \delta (x,y) \end{array} \right] , \end{aligned}$$

and the resulting IMP model no longer has the compactness properties needed to use much of the theory of Lutscher and Lewis (2004). We could either approximate the delta function by a different dispersal kernel with very small variance, as was done by Hardin et al. (1990), or we could use further results of Lutscher and Lewis (2004). Biologically, their general idea is that every individual disperses at some time in its life cycle, and they consider, for some *n*, $\mathcal {F}^n$.

They split the nonlinear operator $\mathcal {F}$ up into the two operators $\mathcal {F}_1$ and $\mathcal {F}_2$, so that $\mathcal {F}_1$ contains all entries where, for every non-zero $r_{ij}$ we have that $k_{ij}(x,y) \in (L^2(\varOmega ))^2$ and is zero everywhere else, and $\mathcal {F}_2$ contains the function $r_{ij}$ where $k_{ij} = \delta (x,y)$ and is zero otherwise. Now $\mathcal {F}_1$ is compact and $\mathcal {F}_2$ is bounded and by Lemma 8 in Lutscher and Lewis (2004), if $\mathcal {F}_2$ is nilpotent of order *n*, $\forall y \in \varOmega$, then $\mathcal {F}^n$ is completely continuous and this allows us to study the existence of fixed points of $\mathcal {F}^n$. Note that a fixed point of $\mathcal {F}^n$ corresponds to a periodic solution, but if there exists a solution which is both a fixed point of $\mathcal {F}^n$ and $\mathcal {F}^{n+1}$, then it is the unique fixed point of $\mathcal {F}$ (Lutscher and Lewis 2004).

Lutscher and Lewis (2004) have also shown the existence of a positive fixed point stemming from a transcritical bifurcation at a critical domain length or growth rate and under the assumptions that the spectral radius of $\mathcal {F}'(\infty )\, \mathrm{is\, less\, than}\, 1$ and that the dominant eigenvalue of $\mathcal {F}'(0)$ is greater than 1. The positive fixed point is unique if $\mathbf {R}(\mathbf {N}(y))$ is monotonic increasing (i.e. for Beverton–Holt type functions, but not Ricker or logistic) and is density dependent with diminishing returns for all values of $\mathbf {N}$ (i.e. no Allee effects). In the event that the concavity condition above is not met, we may see periodic solutions or even chaotic ones (Lutscher and Lewis 2004).

#### Stage-Structured Population Example

We illustrate the bifurcation structure of the example outlined in Sect. 4.2.2 by looking at the linearization around the trivial zero steady state. This results in the eigenvalue problem

87 $$\begin{aligned} \lambda \pmb {\phi }(x) = \int _{-L/2}^{L/2}{ \left( \left[ \begin{array}{cc} \delta (x,y) &{} \frac{1}{2b}\mathrm{e}^{-\frac{1}{b}|x-y|} \\ \delta (x,y) &{} \delta (x,y) \end{array} \right] \circ \left[ \begin{array}{cc} 0 &{} r \\ \alpha &{} \beta \end{array}\right] \right) \left[ \begin{array}{cc} \phi _1(y) \\ \phi _2(y) \end{array} \right] \mathrm{d}y }, \end{aligned}$$

where $b = \sqrt{D/\alpha }$ and $\pmb {\phi } = [\phi _1,\, \phi _2]^\mathrm{T}$. Continuing to follow the ideas of Lutscher and Lewis (2004) and building on the scalar case presented by Kot and Schaffer (1986), we differentiate twice and examine the relationship between the demographic parameters and the domain length. Differentiating (87) with respect to *x* yields

88a $$\begin{aligned} \phi _1'(x) =&-\left( \frac{r}{2\lambda b^2}\right) \mathrm{e}^{-x/b}\int _{-L/2}^{x}{\mathrm{e}^{y/b}\phi _2(y)\mathrm{d}y} \nonumber \\&+\left( \frac{r}{2\lambda b^2}\right) \mathrm{e}^{x/b}\int _{x}^{L/2}{\mathrm{e}^{-y/b}\phi _2(y)\mathrm{d}y} \end{aligned}$$

88b $$\begin{aligned} \phi _2'(x) =&\left( \frac{\alpha }{\lambda - \beta }\right) \phi _1'(x), \end{aligned}$$

and differentiating again results in

89a $$\begin{aligned} \phi _1''(x) =&\frac{-r}{\lambda b^2}\phi _2(x) + \frac{1}{b^2}\phi _1(x),\end{aligned}$$

89b $$\begin{aligned} \phi _2''(x) =&\frac{\alpha }{\lambda -\beta }\phi _1''(x), \end{aligned}$$

with boundary conditions

89c $$\begin{aligned} \pmb {\phi }'(x)&= \frac{1}{b}\pmb {\phi }(x), \quad x = -L/2, \end{aligned}$$

89d $$\begin{aligned} \pmb {\phi }'(x)&= -\frac{1}{b}\pmb {\phi }(x), \quad x = L/2. \end{aligned}$$

To begin to find a solution to this boundary value problem, Lutscher and Lewis (2004) assume solutions of the form $\phi _1(x) = A_1 \mathrm{e}^{\sigma x}$ and $\phi _2(x) = A_2 \mathrm{e}^{\sigma x}$ for some $A_{1,2}$ and $\sigma$, resulting in the system of equations

90a $$\begin{aligned} A_1 \sigma ^2&= \frac{-r}{\lambda b^2} A_2 + \frac{1}{b^2}A_1 \end{aligned}$$

90b $$\begin{aligned} A_2 \sigma ^2&= \frac{\alpha }{\lambda - \beta }A_1\sigma ^2. \end{aligned}$$

In the event that $\sigma ^2 = 0$, we have the relationship

91 $$\begin{aligned} A_2 = \left( \frac{\lambda }{r}\right) A_1, \end{aligned}$$

and so our first set of solutions is

92a $$\begin{aligned} \phi _1&= A_1\end{aligned}$$

92b $$\begin{aligned} \phi _2&= \left( \frac{\lambda }{r}\right) A_1. \end{aligned}$$

If $\sigma ^2 \ne 0$, we obtain another relationship between the constants, namely

93 $$\begin{aligned} A_2 = \left( \frac{\alpha }{\lambda -\beta }\right) A_1. \end{aligned}$$

Using this (and the assumption that $A_1 \ne 0$), we find

94 $$\begin{aligned} \sigma _{\pm } = \pm \sqrt{\left( \frac{1}{b^2}\right) \left( 1 - \frac{r\alpha }{\lambda (\lambda - \beta )}\right) } \end{aligned}$$

and obtain two more solutions

95a $$\begin{aligned} \phi _1&= A_+ \mathrm{e}^{\sigma _+ x} + A_- \mathrm{e}^{\sigma _- x}, \end{aligned}$$

95b $$\begin{aligned} \phi _2&= \left( \frac{\alpha }{\lambda -\beta }\right) A_+ \mathrm{e}^{\sigma _+ x} + \left( \frac{\alpha }{\lambda -\beta }\right) A_- \mathrm{e}^{\sigma _- x}. \end{aligned}$$

We look now for a fourth solution by assuming a different solution form, $\phi _1(x) = B_1 x$ and $\phi _2 (x) = B_2 x$. Since $\phi _1'' = \phi _2'' = 0$, we obtain the relationship

96 $$\begin{aligned} B_2 = \left( \frac{\lambda b^2}{r}\right) B_1, \end{aligned}$$

which results in our fourth set of solutions,

97a $$\begin{aligned} \phi _1&= B_1 x \end{aligned}$$

97b $$\begin{aligned} \phi _2&= \left( \frac{\lambda b^2}{r}\right) B_1 x. \end{aligned}$$

Any linear combination of these is also a solution, and putting them together results in a general set of solutions of the form

98a $$\begin{aligned} \phi _1&= A_1 + B_1 x + A_+\mathrm{e}^{\sigma _+ x} + A_- \mathrm{e}^{\sigma _- x} \end{aligned}$$

98b $$\begin{aligned} \phi _2&= \left( \frac{\lambda }{r}\right) A_1 + \left( \frac{\lambda b^2}{r}\right) B_1 x + \left( \frac{\alpha }{\lambda - \beta }\right) A_+\mathrm{e}^{\sigma _+ x} + \left( \frac{\alpha }{\lambda -\beta }\right) A_-\mathrm{e}^{\sigma _- x}, \end{aligned}$$

and we can now solve for the four unknowns, $A_1$, $B_1$, $A_+$, and $A_-$, by using the four boundary conditions on $\phi _1'(x)$ and $\phi _2'(x)$ at $x = \pm L/2$. In order for there to exist a non-trivial solution to this system of equations, the following condition must be satisfied:

99 $$\begin{aligned}&\left( \frac{2}{b}\right) \left( 1 + \frac{L}{2b}\right) \left[ \left( \frac{1}{b}-s\right) ^2 \mathrm{e}^{-sL} - \mathrm{e}^{sL}\left( \frac{1}{b}+s\right) ^2 \right] \, \nonumber \\&\quad \times \left( \frac{\lambda b^2}{r} - \frac{\alpha }{\lambda -\beta }\right) \left( \frac{\alpha }{\lambda - \beta } - \frac{\lambda }{r}\right) = 0 \end{aligned}$$

where

100 $$\begin{aligned} s = \sqrt{\left( \frac{1}{b^2}\right) \left( 1 - \frac{r\alpha }{\lambda (\lambda - \beta )}\right) }. \end{aligned}$$

We lose the linear stability of the zero steady state as the dominant eigenvalue $\lambda$ passes through $\lambda = 1$, and this leaves us with either

101 $$\begin{aligned}&\left[ \left( \frac{1}{b}-s\right) ^2 \mathrm{e}^{-sL} - \mathrm{e}^{sL}\left( \frac{1}{b}+s\right) ^2 \right] = 0, \end{aligned}$$

102 $$\begin{aligned}&\left( \frac{b^2}{r} - \frac{\alpha }{1-\beta }\right) = 0, \end{aligned}$$

or

103 $$\begin{aligned} \left( \frac{\alpha }{1 - \beta } - \frac{1}{r}\right) = 0, \end{aligned}$$

where *s* is now given by

104 $$\begin{aligned} s = \sqrt{\left( \frac{1}{b^2}\right) \left( 1 - \frac{r\alpha }{1 - \beta }\right) }. \end{aligned}$$

If (101) holds, then we consider two different cases pertaining to the value of *s*. If

105 $$\begin{aligned} 1 - \frac{r\alpha }{1-\beta } > 0, \end{aligned}$$

then *s* is real and $0 < s < 1/b$ in which case

106 $$\begin{aligned} \mathrm{e}^{sL} = \frac{\left( \frac{1}{b} - s\right) }{\left( \frac{1}{b}+s\right) } < 1, \end{aligned}$$

and so *L* must be negative, which is not possible. The other possibility is that *s* is complex (we will address the case when $s=0$ later), i.e. that

107 $$\begin{aligned} 1 - \frac{r\alpha }{1-\beta } < 0. \end{aligned}$$

In this case, *s* is of the form $s = ai/b$, where

108 $$\begin{aligned} a = \sqrt{\frac{r\alpha }{1-\beta }-1}, \end{aligned}$$

and

109a $$\begin{aligned} \mathrm{e}^{\frac{2aiL}{b}}&= \frac{\left( \frac{1}{b} - \frac{ai}{b}\right) ^2}{\left( \frac{1}{b} + \frac{ai}{b}\right) ^2} \end{aligned}$$

109b $$\begin{aligned}&= \frac{1 - 6a^2 + a^4}{(1+a^2)^2} + \frac{4a(a^2-1)}{(1+a^2)^2}i \end{aligned}$$

109c $$\begin{aligned}&= \cos A + i\sin A, \end{aligned}$$

where

110 $$\begin{aligned} A = \arctan {\left( \frac{4a(a^2-1)}{1-6a^2 + a^4}\right) }. \end{aligned}$$

It is important to note that in the event that either $\cos {A} < 0$ or $\sin {A} < 0$, we need to add $\pi$ on to the calculation of $\arctan$ in order to ensure that we obtain a value for *A* in the correct quadrant, i.e.

111 $$\begin{aligned} A = \arctan {\left( \frac{4a(a^2-1)}{1-6a^2 + a^4}\right) } + \pi . \end{aligned}$$

Now we have that

112 $$\begin{aligned} \mathrm{e}^{\frac{2aiL}{b}} = \mathrm{e}^{A i} = \mathrm{e}^{(A + 2n\pi )i}, \end{aligned}$$

and so the critical domain size $L^*$ is

113 $$\begin{aligned} L^* = (A + 2n\pi )\frac{b}{2a} \end{aligned}$$

as in (36). In the special case where $s = 0$ when $\lambda = 1$, (103) is satisfied, and in this way we have infinitely many solutions for the critical domain length. In order to avoid this, we assume a different form for our solutions $\phi _1$ and $\phi _2$, namely

114a $$\begin{aligned} \phi _1&= A_1 + B_1x + C_1x^2 + D_1x^3, \end{aligned}$$

114b $$\begin{aligned} \phi _2&= A_2 + B_2x + C_2x^2 + D_2x^3. \end{aligned}$$

By putting the above two equations into the system of second-order ODEs, we obtain

115a $$\begin{aligned} A_2&= \left( \frac{1}{r}\right) A_1 - \left( \frac{2 b^2}{r}\right) C_1, \end{aligned}$$

115b $$\begin{aligned} B_2&= \left( \frac{1}{r}\right) B_1 - \left( \frac{3 b^2}{r}\right) D_1,\ \end{aligned}$$

115c $$\begin{aligned} C_2&= \frac{\alpha }{1-\beta }C_1, \end{aligned}$$

115d $$\begin{aligned} D_2&= \frac{\alpha }{1-\beta }D_1, \end{aligned}$$

so that the solutions in this special case are of the form

116a $$\begin{aligned} \phi _1 =&A_1 + B_1x + C_1x^2 + D_1x^3, \end{aligned}$$

116b $$\begin{aligned} \phi _2 =&\left( \frac{1}{r}\right) A_1 - \left( \frac{2 b^2}{r}\right) C_1 + \left[ \left( \frac{1}{r}\right) B_1 - \left( \frac{3 b^2}{r}\right) D_1\right] x \nonumber \\&+ \left[ \frac{\alpha }{1-\beta }C_1\right] x^2 + \left[ \frac{\alpha }{1-\beta }D_1\right] x^3. \end{aligned}$$

Applying the four boundary conditions, we obtain four equations in four variables, $A_1$, $B_1$, $C_1$, and $D_1$, and in order for there to exist a unique solution, either

117 $$\begin{aligned} \left( \frac{L^2}{4b}+L\right) \left( \frac{\alpha }{1-\beta }-\frac{1}{r}\right) -\frac{2b}{r} = 0 \end{aligned}$$

which requires $-2b/r = 0$ (since when $s = 0$, $\alpha /(1-\beta ) = 1/r$), which means the population must have a mean dispersal distance of $b = 0$, which is not relevant, or

118 $$\begin{aligned} \frac{3b}{r}\left( b + \frac{L}{2}\right) + \left( \frac{1}{r} - \frac{\alpha }{1-\beta }\right) \left( \frac{L^3}{8b}+\frac{3L^2}{4}\right) = 0 \end{aligned}$$

which requires *L* to be negative and so this solution is also not biologically relevant.

### Example in an Advective Environment

We again attempt to find the dominant eigenvalue of the nonlinear integral operator and use its bifurcation behaviour to determine the critical domain size for the example found in Sect. 4.3.1. Following the same method as in the previous sections, we look at the linearization around the trivial steady state, which results in the following eigenvalue problem

119 $$\begin{aligned} \lambda \mu (x) = \int _{-L/2}^{L/2}{k(x,y)\left[ \frac{\partial f(N(y))}{\partial N(y)}\bigg |_{N\equiv 0}\right] \mu (y)\mathrm{d}y}. \end{aligned}$$

Looking at the linearized IDE, we have

120 $$\begin{aligned} \lambda \mu (x) = r\int _{-L/2}^{L/2} k(x,y)\mu (y)\mathrm{d}y. \end{aligned}$$

Recall that the population can persist if the dominant eigenvalue $\lambda$ of (120) is greater than 1. For a given *r*, the critical domain size is then the value of *L* for which $\lambda = 1$. In this carefully chosen example with the asymmetric Laplace kernel (Table 2), we follow Lutscher et al. (2010) and differentiate (120) twice to obtain a second-order ordinary differential equation with boundary conditions. Using the characteristic equation and letting $\lambda = 1$, we calculate the critical domain size $L^*$ as

121 $$\begin{aligned} L^* = \frac{4\arctan {\left( \sqrt{\frac{4ra_1|a_2|}{(a_1-a_2)^2}-1}\right) ^{-1}}}{(a_1-a_2)\sqrt{\frac{4ra_1|a_2|}{(a_1-a_2)^2}-1}}, \end{aligned}$$

as in (37).
